# Ordered Heterostructured Aerogel with Broadband Electromagnetic Wave Absorption Based on Mesoscopic Magnetic Superposition Enhancement

**DOI:** 10.1002/advs.202301599

**Published:** 2023-05-07

**Authors:** Haojie Jiang, Lei Cai, Fei Pan, Yuyang Shi, Jie Cheng, Yang Yang, Zhong Shi, Xiaoli Chai, Hongjing Wu, Wei Lu

**Affiliations:** ^1^ Shanghai Key Lab. of D&A for Metal‐Functional Materials School of Materials Science & Engineering Tongji University Shanghai 201804 P. R. China; ^2^ Shanghai Key Laboratory of Special Artificial Microstructure Materials and Technology School of Physics Science and Engineering Tongji University Shanghai 201804 P. R. China; ^3^ State Key Laboratory of Pollution Control and Resource Reuse College of Environmental Science and Engineering Tongji University 1239 Siping Road Shanghai 200092 P. R. China; ^4^ MOE Key Laboratory of Material Physics and Chemistry under Extraordinary School of Physical Science and Technology Northwestern Polytechnical University Xi'an Xi'an 710072 P. R. China

**Keywords:** electromagnetic coupling, increased permeability, low‐frequency migration, magnetic‐ordered heterostructure, *N*‐doped MXene aerogels

## Abstract

Demand for lightweight and efficient electromagnetic wave (EW) absorbers continues to increase with technological advances in highly integrated electronics and military applications. Although MXene‐based EW absorbers have been extensively developed, more efficient electromagnetic coupling and thinner thickness are still essential. Recently, ordered heterogeneous materials have emerged as a novel design concept to address the bottleneck faced by current material development. Herein, an ordered heterostructured engineering to assemble Ti_3_CNT_x_ MXenes/Aramid nanofibers/FeCo@SiO_2_ nanobundles (FS) aerogel (AMFS‐O) is proposed, where the commonly disordered magnetic composition is transformed to ordered FS arrays that provide more powerful magnetic loss capacity. Experiments and simulations reveal that the anisotropy magnetic networks enhance the response to the magnetic field vector of EW, which effectively improves the impedance matching and makes the reflection loss (RL) peaks shift to lower frequencies, leading to the thinner matching thickness. Furthermore, the temperature stability and excellent compressibility of AMFS‐O expand functionalized applications. The synthesized AMFS‐O achieves full‐wave absorption in X and Ku‐band (8.2–18.0 GHz) at 3.0 mm with a RL_min_ of −41 dB and a low density of 0.008 g cm^−3^. These results suggest that ordered heterostructured engineering is an effective strategy for designing high‐performance multifunctional EW absorbers.

## Graphical abstract

Benefiting from the enhanced response of the novel magnetic nanobundles and magnetic‐ordered structure to the magnetic field vector of electromagnetic waves, which balances the dielectric loss generated by the MXene network, the MXene based magnetically ordered heterogeneous structured aerogel material constructed in this work has excellent electromagnetic wave absorption performance covering *X* and *Ku* bands at low density.

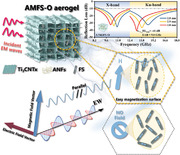

## 1 Introduction

Nowadays, the proliferation of electronic devices and extension of operation frequencies improve the demands for electromagnetic wave absorption (EWA) materials in both the military and civilian sectors.^[^ ^1^ ^]^ Meanwhile, the integration of structure and function trends put forward more stringent requirements for lightweight EW absorbers. Therefore, porous aerogel materials assembled with nanomaterials receive intensive attention as multifunctional EWA. As the least dense solid in the world, aerogels possess high porosity, large specific surface area, large interconnection networks and 3D hierarchical structures, which is beneficial to achieve lightweight and high‐efficient EW absorbers.^[^ ^2^ ^]^

Recently, a new type of 2D metal carbides and nitrides, so‐called MXenes (general formula: M_n+1_X_n_T_x_),^[^ ^3^ ^]^ with exceptional metal‐like conductivity, abundant surface terminations, and lamellar structure,^[^ ^4^ ^]^ is believed to be a promising candidate for EWA applications. Whereas, the intrinsic self‐restacking issue and excessively high conductivity further hinder the application of MXene‐based EWA materials.^[^ ^5^ ^]^ Therefore, the design of a heterogeneous aerogel structure (e.g., composited with carbon materials,^[^ ^6^ ^]^ metal sulfides,^[^ ^7^ ^]^ cellulose^[^ ^8^ ^]^) is put forward to enhance polarization and enlarge the interlayer spacing of the original MXene. However, MXenes cannot meet the need for excellent EWA with thinner thickness due to the lack of magnetic loss.^[^ ^9^ ^]^ To overcome the issue, various magnetic modifications on MXene have been proposed, including MXene/rGO/Ni aerogel,^[^ ^10^ ^]^ MXene/rGO/FeS foam,^[^ ^11^ ^]^ MXene/ultra‐long Ni Chains aerogel.^[^ ^12^ ^]^ Although many magnetic components have been developed with good results,^[^ ^13^ ^]^ the traditional preparation methods are still energy‐consuming and time‐consuming. Furthermore, for the reported magnetic MXene composites, the magnetic components principally exhibit random and disordered arrangements resulting in the diminution of the complex permeability, which is unfavorable for the enhancement of magnetic loss capacity. According to the transmission line theory in Equations 1 and 2, higher permeability is desired in good impedance matching for designing thin absorbers, but the simple hybrid design of a traditional disordered heterogeneous structure falls short of the requirement. Therefore, new structural design strategies need to be developed.

Ordered heterostructured materials (OHM) refer to materials composed of constituent primitives that are ordered on one or more scales, leading to improved material properties.^[^ ^14^ ^]^ OHM has shown great progress in the fields of thermoelectric materials,^[^ ^15^ ^]^ catalytic materials,^[^ ^16^ ^]^ and battery materials,^[^ ^17^ ^]^ highlighting the universality of this concept. Notably, ordered magnetic chains have been demonstrated to further enhance microwave magnetic permeability and magnetic loss.^[^ ^18^ ^]^ This effect is attributed to the alignment of the easy magnetization planes of the ordered particles along the same basal plane, rendering them highly susceptible to magnetization.^[^ ^19^ ^]^ Thus, the combination of dielectric‐lossy and magnetic‐lossy materials by ordered heterostructured design can produce ordered electromagnetic heterogeneous interfaces. However, the reported packaging of ordered magnetic chains is almost insulating material (epoxy,^[^ ^18b^ ^]^ PVDF,^[^ ^18c^ ^]^ paraffin^[^ ^20^ ^]^), which causes the lack of dielectric loss. As far as we know, no research has been reported for the construction of ordered magnetic structures in flexible and conductive aerogel frames. Therefore, it is worth studying to construct aerogel‐based OHM to improve EWA property.

In response to the challenges, a unique magnetic‐ordered heterostructured aerogel has been designed. The conductive skeleton of the aerogel is *N*‐doped MXene (Ti_3_CNT_x_), which exhibited anomalous EW absorption compared to its more conductive Ti_3_C_2_T_x_ in the previous research.^[^ ^21^ ^]^ ANF with strong mechanical strength and thermal stability is used as a high‐performance polymer substrate for aerogel. To construct strong magnetic ordered arrays, we propose “Magnetic field induced pneumatic atomization reduction” (MPAR) to fabricate FeCo@SiO_2_ nanobundles (FS). The ordered arrays of strongly magnetic chains were introduced in isotropic ANFs/MXene aerogel by the external directional magnetic field. The as‐fabricated ANFs/Ti_3_CNT_x_ MXene/FS aerogel (AMFS‐O) shows several notable advantages for EWA.

First, the construction of effective MXene conductive networks with extensive hydrogen‐bonding interactions leads to synergistic effects of intense interfacial polarization and electrical conductivities. Second, the construction of ordered electromagnetic heterogeneous interfaces improves impedance matching. Third, ordered FS arrays enhance the response to the magnetic vector of EW, which significantly promotes the improvement of magnetic loss capacity. Hence, the synthesized AMFS‐O exhibits satisfactory EWA performance covering the X‐band (8.2–12.4 GHz, the most common radar frequency range) and RL_min_ of −40 dB at 3.0 mm with a low density of 0.008 g cm^−3^. This work provides an efficient strategy to maximize the merits of magnetic components, optimizing matching thickness and absorbing properties.

## 2 Results and Discussion

### 2.1 Designing of Ordered Structure Based on the Ferromagnetic Units in ANFs/MXenes Aerogel

**Figure 1:**
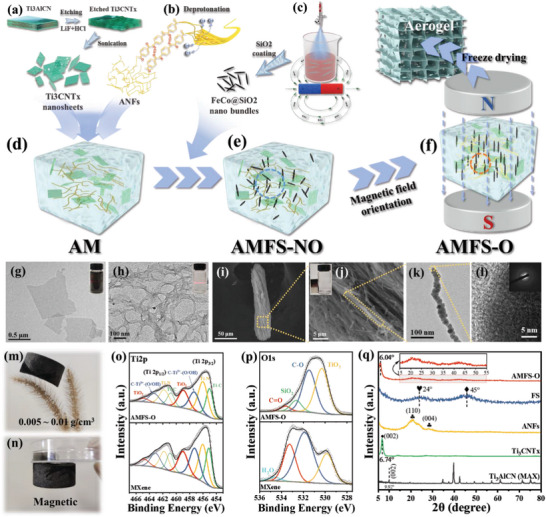
Schematic of preparation process of a) Ti_3_CNT_x_ MXene from Ti_3_AlCN MAX, b) ANFs exfoliated from aramid fibers, c) FeCo@SiO_2_ nanobundles (FS). The homogeneous aqueous dispersion of d) ANFs/Ti_3_CNT_x_ (AM), e) magnetic disordered ANFs/Ti_3_CNT_x_/FS nanobundles (AMFS‐NO), f) magnetic ordered ANFs/Ti_3_CNT_x_/FS (AMFS‐O). TEM images of g) Ti_3_CNT_x_ nanosheets and h) ANFs. i,j) SEM images of prepared single FS nano bundle. k) TEM, and l) HRTEM images of a single FeCo nanowire. The optical photograph of m) AMFS‐O aerogel placed on top of a Setaria Viridis and n) attracted by a magnet. Comparison of o) Ti 2p and p) O 1s high‐resolution XPS spectra of AMFS‐O and Ti_3_CNT_x_. q) XRD patterns of FS, ANFs, Ti_3_CNT_x_ MXene, Ti_3_AlCN MAX, and A7M3FS‐O.

The micron‐scale ordered heterostructured AMFS was prepared by the ice template method as revealed in **Figure** **1**. Ti_3_CNT_x_ nanosheets, ANFs, and FeCo@SiO_2_ nanobundles were separately prepared and assembled. Figure 1a shows the synthesis of Ti_3_CNT_x_ MXene with HCl/LiF by selectively etching the Al layers from Ti_3_AlCN MAX powder. The obtained multilayer Ti_3_CNT_x_ exhibits an accordion‐like structure (Figure S2, Supporting Information) and the ultrathin and transparent MXene nanosheets can be observed from TEM images (Figure 1g). A highly stable and homogeneous ANFs dispersion was fabricated by deprotonation of ultra‐strong para‐aramid fiber (Kevlar 29, Figure 1b).^[^ ^22^ ^]^ As shown in Figure 1h and Figure S3 (Supporting Information), the diameter of the original aramid fiber is ≈12 µm and the obtained ANFs aqueous dispersion presents a radial size of 20 nm. The obtained Ti_3_CNT_x_ MXene dispersions (Figure 1g, inset) and ANFs in aqueous dispersion (Figure 1h, inset) all have a strong Tyndall effect, indicating the colloidal characteristic and good dispersibility.

Figure 1c displays the preparation of FeCo@SiO_2_ nanobundles (FS). Distinct from traditional methods of magnetic field heat treatment^[^ ^18c^ ^]^ or titration,^[^ ^23^ ^]^ which causes high energy and time consumption, we propose “Magnetic field induced pneumatic atomization reduction” (MPAR) to fabricate nanoscale ordered FeCo nanobundles. Numerous FeCo nanowires are converged in the same direction to form highly anisotropic FeCo nanobundles. Typically, the reducing agent (NaBH_4_) was injected into the metallic salt solution by rapid gas atomization with a low‐pressure pneumatic spray gun. The reaction takes place as Equation S2 (Supporting Information). In the simple magnetic field created by a permanent magnet, the nano‐scale ferromagnetic metal particles reduced in the solution were instantly connected and self‐assembled into a 1D magnetic chain arranged in the direction of the magnetic field, thereby forming ultra‐long nanowires. During the whole reaction process, the in situ generated hydrogen protected the nanowires from oxidation. Moreover, each nanowire was further extended along with the long chains of polyvinylpyrrolidone (PVP) as a template in the solution. Meanwhile, the surface activity of the nanowires was improved by the PVP, so that the nanowires were adsorbed one by one and assembled in parallel to form nanobundles in the magnetic field. The SEM images of a nanobundle are shown in Figure 1i,j, the length, and the diameter of which can be 200 and 30 µm respectively. It is different from the nanowires prepared without PVP in Figure S5a (Supporting Information). Whereafter, SiO_2_ was coated on the prepared nanobundles to prevent oxidation using a modified Stöber method,^[^ ^24^ ^]^ of which the reaction can be briefly summarized as Equations S3 and S4 (Supporting Information). EDS images corresponding to Figure 1i exhibit the existences of Fe, Co, Si, and O (Figure S6, Supporting Information), which illustrates the successful synthesis of the FS possessing abundant heterogeneous interfaces. Moreover, the antioxidant effect of SiO_2_ coating is shown in Figure S7 (Supporting Information). The high magnetism enables quick magnetic separation of the nanobundles from their aqueous dispersion (≈1.0 mg mL^−1^) in 10 s using a magnet (≈100 mT, Figure 1j, inset). Furthermore, the single nanowire stripping from FS with an average width of less than 50 nm is manifested from the TEM images (Figure 1k). Combining with the HRTEM images and selected area electron diffraction (SAED) images in Figure 1l, the amorphous structure feature of FS can be primarily confirmed.

To prepare the ultra‐flexible magnetic‐ordered hybrid aerogel, ANFs and Ti_3_CNT_x_ MXene were uniformly mixed in a particular proportion and formed a stable dispersion (AM, Figure 1d) based on the action of hydrogen bonds, which is confirmed by the redshift of N—H and C=O bonds in the FTIR spectra (Figure S8, Supporting Information). Next, FS was added to the ANFs/MXene dispersion and assembled in the liquid phase. Subsequently, the mixture was placed in a parallel magnetic field, causing FS to align along the magnetic field line. Cryogenic freezing was performed on this aligned mixture, followed by freeze drying, resulting in the ultra‐flexible magnetic‐ordered hybrid aerogel, named AMFS‐O (Figure 1f). In contrast, the mixture frozen without magnetic field orientation is tabled as AMFS‐NO (Figure 1e). The AMFS‐O aerogel exhibited characteristics of lightweight (a density of 0.008 g cm^−3^, placed on top of a Setaria Viridis, Figure 1m) and magnetism (Figure 1n).

The chemical structures and elements of the assembled aerogel were then thoroughly investigated. X‐ray photoelectron spectroscopy (XPS) was carried out to demonstrate the surface chemical structures of pristine materials and synthetic aerogel. As can be seen, larger peaks of TiO_2_ in Ti 2p (Figure 1o) and O 1s spectra (Figure 1p) are detected in the composite sample than that in pure MXene. The mild oxidation may result from ultrasound, agitation, and slight dissociative Fe^3+^. Other XPS spectra are displayed in Figure S9 (Supporting Information). The deeper crystallographic structures of samples were detected by X‐ray diffraction (XRD). In contrast to pristine MXene, the introduction of ANFs makes the characteristic peak (002) of AMFS‐O shift from 6.74° to 6.04°, indicating that ANFs have been successfully incorporated into the MXene. Broad peaks indexed to ANFs, and FS is visible in AMFS‐O. These results illustrate that no obvious oxidization of Ti_3_CNT_x_ occurred during the assembly process, accompanied with a favorable combination, implying the successful preparation of ternary composite aerogels, and guaranteeing the final mechanic properties and EWA performance of AMFS aerogels. Optical photographs of the prepared AM aerogels and magnetic‐ordered AMFS‐O aerogel are shown in Figure S10 (Supporting Information).

The sound arrangement of constructed conductive, magnetic networks and the resulting synergetic morphologies in aerogels are critical for achieving superior EWA performance. The crux in obtaining ordered magnetic arrays in aerogels is the degree of orientation of the magnetic material in the precursor aqueous dispersion. However, its challenging to achieve macroscopically directed in high‐viscosity dispersions due to the low saturation flux density (B_s_) and magnetic anisotropy (MA) of common magnetic chains. In contrast, FS consisting of numerous Fe‐Co magnetic chains has enhanced B_s_ and MA, which enables them to break the obstruction of the viscosity by the magnetic field and arrange uniformly along the magnetic lines of force.

**Figure 2:**
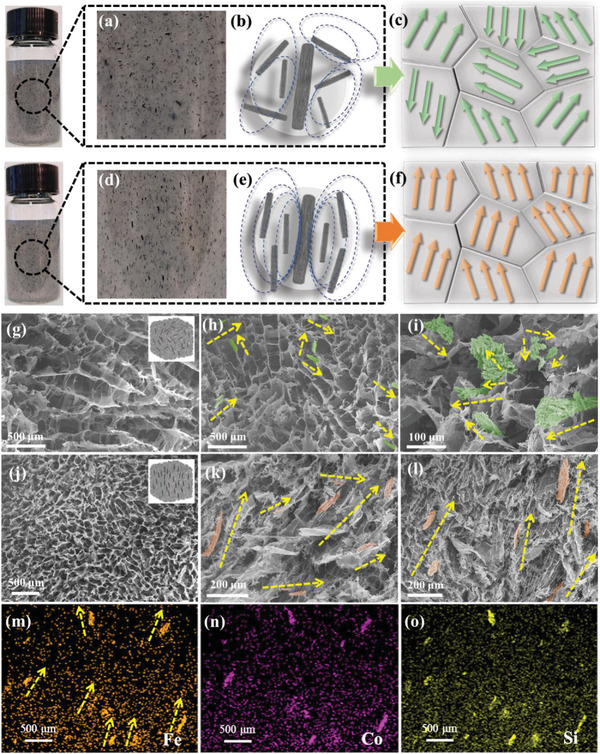
a) Optical photographs of disordered FS in ANFs dispersion. Schematic illustration of b) magnetic coupling and c) “grains” and “crystal” of the disordered aerogel structure. d) Optical photographs of ordered FS in ANFs dispersion. Schematic illustration of e) magnetic coupling and f) “grains” and “crystal” of the ordered aerogel structure. g–i) SEM images of the longitudinal section plane of AMFS‐NO. j–l) SEM images of the longitudinal section plane of AMFS‐O. EDS mapping of m) Fe, n) Co, o) Si corresponding to (j), showing the ordered distribution of FS.

As anticipated, the directed magnetic field caused the FS in the ANFs aqueous dispersion (4 mg mL^−1^) to transition from disordered (**Figure** **2**a) to ordered (Figure 2d) (For clearly observing the dispersion of FS, MXene was not introduced). Therefore, the well arrangement of FS in the precursor aqueous dispersion can be retained in the freeze‐dried aerogel structure, guaranteeing the construction of ordered magnetic network. Since the incurred magnetic fields were aligned in a single direction, the magnetic coupling was strengthened in the ordered configuration (Figure 2e). Conversely, the resulting magnetic fields eventually cancel each other out in the random system, possibly because the magnetic chains are entangled (Figure 2b). To better understand, we consider the spatial domain affected by the magnetic moment of one FS as a “grain”. Since FS is formed by numerous magnetic nanowires aggregated along the same direction, the magnetic moments inside this “grain” can be considered as ordered. Numerous “grains” form the magnetic network resembling “crystals”. In Figure 2c, the macroscopic “crystal” exhibits a weaker magnetic moment due to the partial cancellation of magnetic moments among different “grains”. Therefore, the ability of magnetic responsiveness to EW is insufficient. This phenomenon is similar to that the disordered magnetic moment of soft magnetic materials requires more time to deflect toward the applied magnetic field during initial magnetization, leading to a decrease in permeability.^[^ ^25^ ^]^ The ordered “crystal” depicted in Figure 2f demonstrates macroscopic magnetic anisotropy resulting from the ordered magnetic moment arrangement caused by “grain” orientation, which also reduces the alignment time of the overall magnetic moment under the action of electromagnetic fields, thus improving the magnetic responsiveness.

To confirm the distribution of FS in the aerogels, longitudinal sections of aerogels with magnetic‐disordered (AMFS‐NO, Figure 2g) and magnetic‐ordered (AMFS‐O, Figure 2j) were investigated through SEM. In the framework of aerogels, numerous ANFs networks act as the supporting skeleton. They are benefiting from the hydrogen bond binding between the hydroxyl group on the surface of MXene and the carbonyl group of ANFs, which makes MXene nanosheets uniformly coat on ANFs networks, while effectively preventing the agglomeration at the same time. The AMFS‐NO and AMFS‐O possess typical micro‐porous structures, giving the aerogel lower density and space for the multiple reflections of EMWs. Figure 2h,i displays the FS distribution in the AMFS‐NO networks (green marked for FS) without applied magnetic field during freezing. As can be seen, the angle between most FS is greater than or equal to 90° (Figure 2h), and random FS traverses the observation plane (Figure 2i), which can be considered a disordered state. For comparison, the FS in the AMFS‐O is mainly arranged in parallel or at an acute angle (Figure 2k,l, red marked for FS), which forms a novel highly ordered macroscopic morphology. Because of the parallel magnetic field applied during freezing, the FS is aligned along the magnetic field line, and the magnetic‐ordered structure is preserved during the sublimation of the ice crystal. The energy dispersive spectroscopy (EDS) mapping images (Figure 2m,n) corresponding to Figure 2j confirms that Fe, Co, Si, O, C, N, and Ti elements are present in the AMFS‐O (EDS of O, C, N, and Ti elements were shown in Figure S11, Supporting Information), the prominent colors of Fe, Co, Si elements further reflect the distribution of FS. Remarkably, as‐prepared micron‐scale ordered heterostructured aerogel material can be considered as an ordered electromagnetic structure, thus displaying great potential to achieve superior EWA performance.

### 2.2 Electromagnetic Performance

First, we focused on the study of the EWA property of the two‐component ANFs/MXene (AM) hybrid aerogel to discover the optimal mass ratio as the basis for the final ternary system. XRD patterns of each AM sample are shown in Figure S12 (Supporting Information), which demonstrates the successful combination of ANFs and MXene. Figure S13 (Supporting Information) shows the electromagnetic parameters and horizontal comparison of EWA properties of different AM samples. The real permittivity (*ε*′) and imaginary permittivity (*ε*″) show a typical frequency dispersion phenomenon. The *ε*′ of AM decreases from 10.59 (A5M5) to 3.00 (A8M2) when the addition of Ti_3_CNT_x_ decreases. According to the effective medium theory and free electron theory (*ε*′′ ≈ *σ*/(2*πε*_0_ *f*, where *σ* is electrical conductivity),^[^ ^26^ ^]^ the decreased *ε*′ and *ε*″ can be ascribed to the reduction of electron transmission and conduction loss caused by decreasing the ratio of high conductive MXene in the networks. By comparison, the µ′ and µ″ are stable because none of the magnetic substances was added. In general, the relevant frequency band with RL < 10 dB is regarded as an effective absorption bandwidth (EAB). Therein, the EAB of A7M3 reaches 2.88 GHz and the strongest reflection loss (RL_min_) is −11.6 dB at a thickness of 3.3 mm, which is better than other components. Therefore, the A7M3 with optimal EW attenuation ability was selected as the foundation to construct AMFS. However, it should be noted that even for A7M3, the EAB and matching thickness are still unsatisfactory. The reason is that the single conduction loss is incapable of producing sufficient dissipation capacity for low‐frequency EW and other loss forms are lacking at the same time.

**Figure 3:**
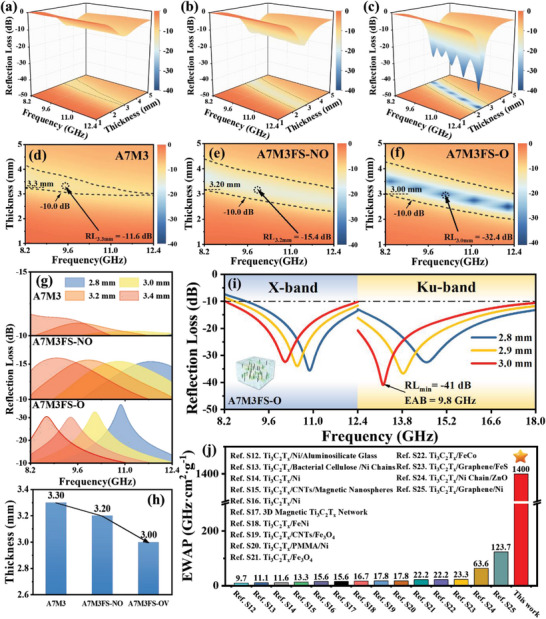
The 3D plots of RL values versus frequency and thickness of a) A7M3, b) A7M3FS‐NO, c) A7M3FS‐O. The 2D contours of RL values versus frequency and thickness of d) A7M3, e) A7M3FS‐NO, f) A7M3FS‐O. g) RL curves (below −10 dB) for all samples within a thickness range of 2.8–3.4 mm. h) Comparison chart of matching thickness decreasing with increasing degree of order. i) RL curves of A7M3FS‐O at 2.8, 2.9, 3.0 mm from 8.2–18 GHz. j) Comparison of the EWA properties of A7M3FS‐O in this work with the reported magnetic MXene‐based EWA materials in X‐band.

The EWA performances of AMFS aerogels are evaluated by the wave‐guide method, which ensures the integrity of porous and magnetic‐ordered structures. Samples were cut to the size of the test mold cavity for insertion (Figure S14, Supporting Information). The 3D plots of RL values versus frequency and thickness of all samples are shown in **Figure** **3**a–c, corresponding to 2D RL contours of Figure 3d–f, respectively. Briefly, the EWA performances (EAB and RL value) are improved significantly with the addition of FS and ordering. More detailed RL curves with different thicknesses at X‐band are shown in Figure S15 (Supporting Information). Figure 3g demonstrates the RL curves of all samples at a thickness of 2.8–3.4 mm. Interestingly, the RL_min_ peaks at each thickness of A7M3FS‐O shift to lower frequencies compared with that of A7M3FS‐NO and A7M3. In other words, the matching frequencies of the ordered composite are lower than those of the random one at the same thickness. Moreover, the EAB of both magnetic‐ordered and random samples can cover the entire X‐band at 3.2 and 3.0 mm, respectively, but the RL_min_ values of A7M3FS‐O is much higher than that of A7M3FS‐NO.

In practical applications, a smaller thickness of MAs is an ambition that has always been pursued. It is visible that the matching thickness with best EAB decreases from 3.30 to 3.00 mm after orientation (Figure 3h). The enhancement of EWA performance can be seen as a superimposed strengthening resulting from the magnetic ordering. The ordered sample further enhances the reflection loss and reduces the matching thickness while keep the whole X‐band absorption of the disordered sample. These results indicate that our ordered AMFS‐O aerogels can be used as thinner microwave absorbers for their novel microwave properties. To verify the broadband absorption of A7M3FS‐O, we extend the test band to Ku‐band. The RL curve of A7M3FS‐O from 8.2 to 18.0 GHz is displayed in Figure 3i and the related electromagnetic parameters are shown in Figure S16 (Supporting Information). As can be seen, the A7M3FS‐O's EAB can cover the X‐band and Ku‐band up to 9.8 GHz with a matching thickness of 3.0 mm.

It is worth noting that absorption ability and bandwidth can be efficiently adjusted by electromagnetic coupling and ordered heterogeneous engineering. For a more comprehensive evaluation, the specific EWA performance (EWAP), which includes EAB, density, and thickness simultaneously, is used to evaluate the samples. The EWAP of A7M3FS‐O aerogels is reached to 1400 GHz cm^2^ g^−1^. Besides, other magnetic MXene‐based EWA materials in X‐band are summarized and their corresponding EWAP is shown in Figure 3j and Table S1 (Supporting Information). Conventional high filling ratio (mixed with adhesive) electromagnetic MXene‐based materials still need to improve the absorption efficiency, and even aerogel materials cannot perform structurally complete integrated EWA to achieve lightness and efficiency, so the EWAP values are commonly lower than 200. As a holistic EWA material, AMFS‐O can achieve efficient absorption in X‐band with a low surface density, of which the EWAP is superior to these reported magnetic MXene‐based materials. In addition, the ordered strategy also enables AMFS‐O to have a broader optimization space.

### 2.3 Analysis of Electromagnetic Parameters and MA Mechanisms

**Figure 4:**
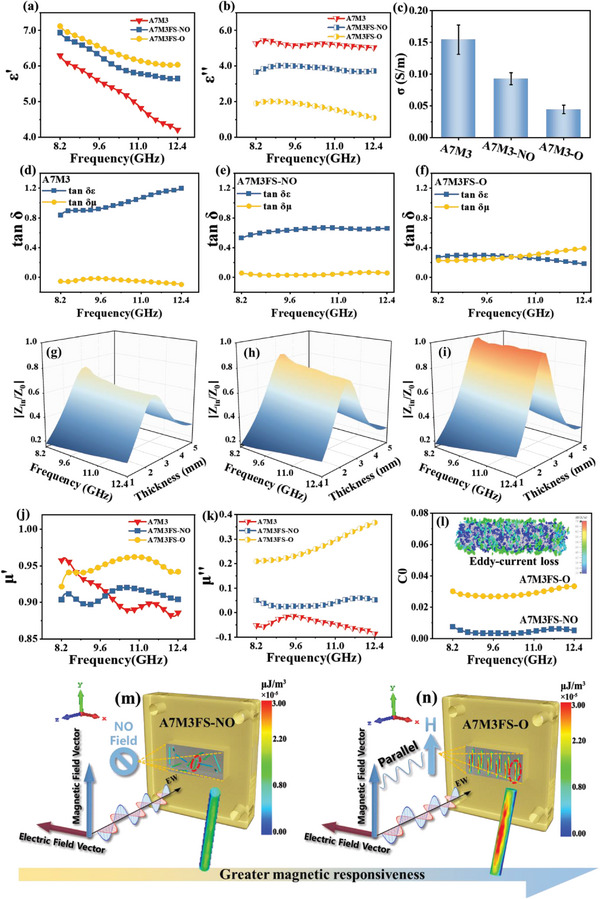
a) The real and b) imaginary part of permittivity of all samples. c) The electrical conductivity of all samples. tan *δ* values of d) A7M3, e) A7M3FS‐NO, f) A7M3FS‐O. Z contours of g) A7M3, h) A7M3FS‐NO, i) A7M3FS‐O. j) The real and k) imaginary part of the permeability of all samples. l) C0 value of A7M3FS‐NO and A7M3FS‐O and surface current simulation of FS. Diagram of sample placement in the waveguide cavity and the magnetic energy density (MED) simulation at 10.3 GHz of m) A7M3FS‐NO. n) A7M3FS‐O.

The effect of ordered heterostructured engineering on the electromagnetic parameters is investigated by complex permittivity (*ε*_*r*_ = *ε*′ − *jε*′′) and permeability (*µ*_*r*_ = *µ*′ − *jµ*′′). Generally, the *ε*′ represents the ability to store charge, which is related to the multi‐polarizations and the *ε*″ represents the ability to attenuate EW energy, which is associated with conductive loss.^[^ ^12^ ^]^ As exhibited in **Figure** **4**a, the value of *ε*′ follows the order of: A7M3FS‐O > A7M3FS‐NO > A7M3, indicating that the ability to store electrical charge and polarization is gradually strengthened with the ordered architecture of functional FS.^[^ ^7b^ ^]^ As to *ε*″ in Figure 4b, the trend is the opposite of *ε*′. Based on the free‐electron theory, the values of *ε*″ are proportional to electrical conductivity (*σ*).^[^ ^26b^ ^]^ The *ε*″ of A7M3 is the highest due to the highest *σ* (Figure 4c), representing the strongest conductive loss. For A7M3FS‐NO samples, the conductive networks of MXenes were interrupted by FS inevitably, causing the decrease of *σ* and *ε*″. Moreover, the *ε*″ of ordered A7M3FS‐O were decreased compared with that of random A7M3FS‐NO, which is consistent with the decreased *σ* in Figure 4c. Furthermore, the decreased trend of *ε*″ is the same as the tan *δε* from Figure 4d to Figure 4f, demonstrating the dominant position of dielectric loss is gradually lost. As in conjunction with Figure 4g–i, the decrease of complex permittivity is beneficial for optimizing the impedance match, which prompts more EW to enter into absorbers for further dissipation.

Notably, higher permeability in the relevant frequency band is required to achieve a thinner and more efficient absorber. The complex permeability (µ′, µ″) of samples is presented in Figure 4j,k. The µ′ is linked to energy storage capability, while the µ″ refers to magnetic loss. The ordered arrangement of FS in a biphasic disordered aerogel enhances the coupling effect between the primitives. Specifically, µ′ and µ″ of the magnetic‐ordered A7M3FS‐O are higher than those of A7M3FS‐NO and A7M3, demonstrating the greater properties of electromagnetic response and magnetic loss. Improved magnetic behaviors of the ordered structure are gradually analyzed as follows:

#### 2.3.1 Enhanced Intrinsic Magnetic Properties Provided by Nanoscale‐ordered FS

Based on the Snoek limit and its modified formulas in the supporting information,^[^ ^27^ ^]^ it can be proved that particles with strong anisotropy and small demagnetization factor in the direction of easy magnetization can obtain better permeability in the GHz frequency band. In this work, the easy magnetization axis of FS is in the axial direction and the demagnetization factor in this direction is zero. FS composed of numerous ordered nanowires exhibits high saturation magnetization (*M_S_* = 196 emu g^−1^, Figure S17, Supporting Information) and significant anisotropy due to the ordering of the arrays, resulting in obvious advantages. Comparing the EWA performance of coaxial rings of FS (Figure S18a, Supporting Information) and FeCo nanowires (Figure S18b, Supporting Information) at a filling ratio of 20 wt.%, it can be observed that FS exhibits a significant performance enhancement, thereby confirming the advantage of nanoscale‐ordered FS as a magnetic network unit. Moreover, carrier transport paths are prone to form in the axial direction under an electromagnetic field due to the natural metallic property of FS, which consumes the energy of EW through conducting loss. CST simulation provides a more intuitive loss mechanism in Figure S19 (Supporting Information), the current along the length of the FS produces a longitudinal superposition effect (blue part) and forms multitudinous toroidal eddy currents (green part) by the alternating electric field, which makes its loss capacity greatly exceed that of the single magnetic chains with only axial current. Thus, the A7M3FS‐O has a higher C_0_ value (*C*_0_ = *µ*′′(*µ*′)^−2^ *f* ^−1^) than that of A7M3FS‐NO representing the stronger eddy current loss throughout the whole X‐band (Figure 4l).

#### 2.3.2 Enhanced Magnetic Response to the Effective Magnetic Field Vector of Incident EW

The enhanced complex permeability of the ordered sample is generally considered to be attributed to the aligned distribution of the ferromagnetic particles in the direction of easy magnetization,^[^ ^20^ ^]^ hence making them highly susceptible to magnetization. The enhancement of electromagnetic response in A7M3FS‐NO and A7M3FS‐O is a result of the interaction between the in‐plane easy magnetization of ordered FS and the effective magnetic field vector of incident EW. The disordered FS in A7M3FS‐NO is not able to exactly match the magnetic field vector of the incident EW in Figure 4m, which thus has a generally weak response of magnetic moments to incident EW. The slight increment in µ′ and µ″ can be ascribed to other in‐plane magnetizations possibly parallel to the magnetic field vector. However, the easy magnetization direction of A7M3FS‐O is parallel to the magnetic field vector of EW, which can effectively increase the response of magnetic moments to microwaves (Figure 4n).^[^ ^28^ ^]^ Furthermore, there are various flip energy thresholds for the magnetic moments at various places when magnetic nanobundles are disordered. Moreover, the maximum angular flip (180 °) of the magnetic moment will occur in magnetic nanobunches when they are aligned parallel to the magnetic field vector of EW, leading to a larger magnetic loss.

#### 2.3.3 Increased Magnetic Energy Density Provided by Mesoscale‐ordered Magnetic FS Arrays

Energy within magnetic materials is a consequence of complex microscopic interactions. Therefore, the magnetic energy density (MED) of A7M3FS‐NO and A7M3FS‐O is simulated by CST to evaluate the magnetic energy stored in FS given by EW for demonstrate the difference in EWA capacity caused by ordered heterostructure (Figure 4m,n). MED refers to the magnetic field energy per unit volume. In the alternating electromagnetic field, the changing electromagnetic field propagates in the form of waves, in which propagates energy. The magnetic field energy can exist apart from the current and the expression of MED is $\psi =\frac{1}{2}\;\mu H^{2}$,^[^ ^29^ ^]^ while the extended information about MED is presented in the supporting information. When a plane wave is transmitted to FS, the magnetization current is generated in the FS and the magnetic field energy in the region is changed. Therefore, the stronger the medium magnetized, the more work the magnetic field does on the medium, and the greater the MED of the medium. From the MED expression, the MED of FS after being magnetized is proportional to the magnetic field strength (H) and its own magnetic permeability (µ). Since the EW frequency in the CST simulation is set at 10 GHz and the location of FS arrays from the plane wave source is fixed, the H at a specific location is treated as constant. Therefore, increased MED implies increased µ. As can be seen, when the FS array is perpendicular to the *z*‐axis, its MED is significantly greater than that of disordered alignment. Correspondingly, higher MED results in high µ, which is consistent with the electromagnetic parameters.

Accordingly, the value of tan *δ*µ and tan *δε* are gradually approaching based on the increased permeability and magnetic loss resulting from ordered heterostructure (Figure 4d–f), which means the change from predominating dielectric loss to balanced electromagnetic loss leading to better impedance matching, as depicted in Figure 4g–i. To sum up, effective interaction between EW and the easy magnetization surface constructed by directional magnetic moment from ordered FS effectively increases the microwave permeability and resonance frequency, promising easier satisfaction of impedance matching as well as decreasing the thickness of the absorber, making the RL_min_ peaks shift to lower frequencies, which explains the phenomenon in Figure 3g.^[^ ^18^, ^30^ ^]^ Based on the above discussion, we are attempting to step outside the confines of the research model of electromagnetic coupling at the nanoscale and to build disordered/ordered magnetic structures at the mesoscopic scale in order to investigate the relationship between structure and properties of heterogeneous structured materials. The superimposed enhancement of dual‐scale (nano and mesoscopic scale) ordered AMFS‐O in balancing electromagnetic losses and enhancing electronic warfare attenuation is verified by theoretical and experimental validation. Therefore, the design of ordered heterostructure has proved to be useful for the design of monolithic EWA materials. Furthermore, more efforts are worth exploring the multi‐scale ordered heterogeneous design to improve the performance of functional materials and generate new functions.

### 2.4 RCS Simulation and Functional Extension

**Figure 5:**
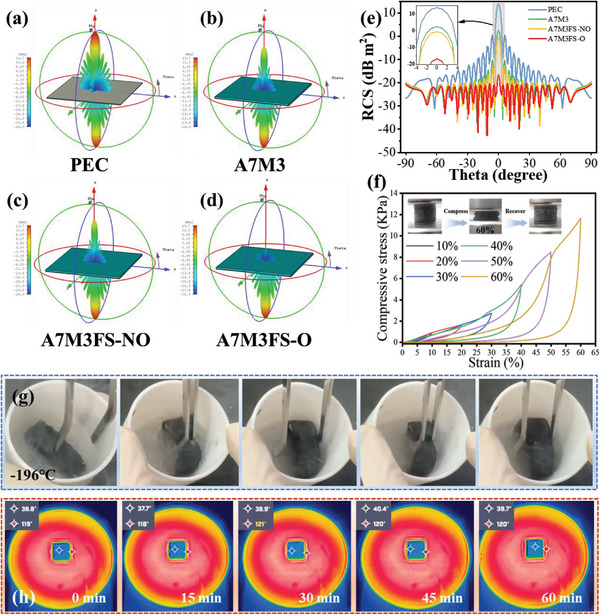
3D RCS plot for the a) PEC substrate covered with b) A7M3, c) A7M3FS‐NO, d) A7M3FS‐O. e) RCS values of all samples within the scanning angle range from −90° to 90°. f) Stress–strain curves of A7M3FS‐O aerogel at compressive strains of 10%, 20%, 30%,40%, 50%, and 60%. g) Compression test of A7M3FS‐O immersed in liquid nitrogen (−196 °C). h) Thermal infrared images of A7M3FS‐O captured at intervals of 15 min from 0 to 60 min.

The radar cross‐section (RCS), a crucial indicator that indicates the actual far field condition EWA performance of the produced materials, must be taken into account in stealth technologies.^[^ ^31^ ^]^ To further prove the microwave stealth function of the prepared aerogel materials, here we simulate the RCS of a PEC (Perfect Electric Conductor, 200 mm×200 mm×2 mm) plate covered with synthesized absorbers under an actual situation. Plane waves were chosen as an excitation source. Three synthesized products were chosen as the coating material, while the coating thickness was set to 3.0 mm. Key equations describing radar and the calculation of RCS are provided in Equations S12 and S13 (Supporting Information).^[^ ^32^ ^]^ As can be observed from the 3D RCS simulation results in **Figure** **5**a–d, A7M3FS‐O exhibits the superb ability to suppress reflected signal and the vertical reflected intensity is much weaker than those of other samples, confirming that more microwave energy can be absorbed by A7M3FS‐O. In the case of all samples, the obtained RCS value is highest at a scanning angle of 0°. The maximum RCS values for the PEC plate and A7M3/A7M3FS‐NO/A7M3FS‐O covered PEC composites are 13.83, 2.48, −0.47, and −16.67 dB m^2^, respectively (Figure 5e). Based on these results, A7M3FS‐O reduces the RCS by 30.5 dB m^2^ compared to plain PEC, further demonstrating the excellent microwave attenuation ability of the A7M3FS‐O aerogel. The highly magnetic ordered structure is responsible for the excellent stealth performance of the material.

Moreover, excellent mechanical resistance and stability are essential for an aerogel‐based EW absorber. The network framework structure and abundant hydrogen bonding interactions between MXene nanosheets and ANF respond cooperatively. Meanwhile, the stout FS provides structural support for the aerogel network, enabling it to external stress and further improve the mechanical performance of AMFS. Figure 5f depicts the typical stress–strain curves of the loading and unloading compression process at strains of 10%, 20%, 30%, 40%, 50%, and 60%. When released from compression at a specified strain even as high as 60%, the A7M3FS‐O aerogel can return to its former shape without obvious structural damage. 11.7 kPa of compressive stress is present at 60% strain. Furthermore, benefiting from the super wide operating temperature and outstanding mechanical properties of ANFs, the A7M3FS‐O aerogel exhibited excellent resilience properties under extremely low‐temperature conditions (liquid nitrogen, −196 °C, Figure 5g), which is significantly better than the rapid failure of other cellulose‐based MXene composites under the same conditions. Such features provide the aerogel with long‐term durability and potential for various applications in extreme conditions. Besides excellent mechanical performance, the aerogel shows excellent thermal insulation properties, which ensures stability in high‐temperature environments. A7M3FS‐O was placed on an enclosed electric furnace heating platform with a set temperature of 120 °C and thermal infrared images were captured from above. The corresponding images after durations of 0 to 60 min are presented in Figure 5h. The temperature at the top of the sample is kept at ≈40 °C despite a long heating process, indicating that the sample has good thermal insulation properties. Considering the application requirements in an actual environment, the magnetic‐ordered heterostructured absorbers promise practical feasibility in both civilian and military stealth applications.

## 3 Conclusion

We have elaborately designed a Ti_3_CNT_x_ MXenes/Aramid nanofibers/FeCo@SiO_2_ nanobundles aerogel with mesoscopic magnetic ordered heterostructure and tunable absorption performance, where the perpendicular through magnetic array network composed of ordered FS arrays significantly improves the response to EW. Based on the interaction between easy magnetization surface and magnetic vector in EW, the MED of ordered FS increases, leading to the increment of the complex permeability and magnetic loss. In addition, the resulting low‐frequency shift of the absorption peaks reduces the matching thickness by 0.2 mm under the same condition. Therefore, the magnetic ordered heterostructure aerogel achieves full‐wave absorption in X‐band and Ku‐band of 3.00 mm with a RL_min_ of −41 dB and the EWAP is higher than 1400 GHz cm^2^ g^−1^ with a density of 0.008 g cm^−3^. Besides, the ordered AMFS‐O aerogel shows excellent compressibility and stable mechanical properties over a wide temperature range from −196 to 120 °C. It should be emphasized here that designing ordered structures to improve the response to EW shows a superposition effect, which means the EWA performance can be further improved based on the original material system by this ordering‐function strategy. With such a contrivable platform, we believe that in‐depth exploitation of ordered heterostructure in EWA engineering would bring more inspiring work.

## 4 Experimental Section

### Chemicals and Materials

Aramid fibers (Kevlar 29) were bought from DuPont Company. Dimethyl sulfoxide (DMSO), potassium hydroxide (KOH), lithium fluoride (LiF), ferric chloride hexahydrate (FeCl_3_·6H_2_O), cobalt chloride hexahydrate (CoCl_2_·6H_2_O), polyvinyl pyrrolidone (PVP, K13 – K18), sodium borohydride (NaBH_4_), tetraethyl orthosilicate (TEOS), concentrated ammonia solution (28 wt %) and ethanol (99.7%) were purchased from Shanghai Aladdin Industrial Corporation (China). Hydrochloric acid (HCl, 36% – 38%) was purchased from Sinopharm Chemical Reagents Co., Ltd. MAX powder (Ti_3_AlCN, 98%) was obtained from Laizhou Kai Xi Ceramic Materials Co. All chemicals without purity identification were of analytical grade without further purification.

### Fabrication of Ti_3_CNT_x_ (MXene)

The typical LiF‐HCl etching method was used to fabricate multilayer Ti_3_CNT_x_. First, 1.0 g of LiF was precisely weighed and then slowly added into 20 mL of HCl solution (12 m) in a Teflon hydrothermal reactor by magnetic stirring for 10 min. Second, 1.0 g of Ti_3_AlCNT_x_ powder was gradually added to the mixed solution and continuously stirred at 50 °C for 24 h. Next, remove the residual LiF in the mixed solution by several centrifugations (3500 rpm, 5 min per wash) until the pH of the supernatant was ≈6. Then, the multilayer Ti_3_CNT_x_ was sonicated in DI water for 1 h to obtain the few‐layered Ti_3_CNT_x_. followed by centrifuging at 4000 rpm for an hour to remove the residual multilayer Ti_3_CNT_x_. Finally, the dark green supernatant containing Ti_3_CNT_x_ nanosheets (12 mg mL^−1^) was gathered and then stored in a refrigerator for further application.

### Fabrication of the Aramid Nanofibers Dispersion

Aramid nanofibers (ANFs) were prepared by Proton Donor‐Assisted Deprotonation according to earlier reported research.^[^ ^33^ ^]^ Briefly, 1.0 g of shortcut fibers, 1.5 g of KOH, and 20 mL DI water were mixed followed by adding 500 mL of DMSO. Then, the Kevlar/KOH/DMSO mixed system was magnetically stirred at room temperature for 4 h evenly under a sealed environment until obtaining a uniform and dark red ANFs dispersion. Subsequently, 1 L of deionized water was injected into the ANF/DMSO dispersion for protonation with constant magnetic stirring. To acquire the purified colloidal ANFs, DI water was used alternately to dissolve away the redundant DMSO and KOH existing in the ANF solution assisted by vacuum filtration until the pH of the dispersion was ≈7. Finally, making the resulting ANFs suspension disperse equably at a concentration of 4 mg mL^−1^.

### Fabrication of FeCo@SiO_2_ Nanobundles (FS)

It was proposed “Magnetic field induced pneumatic atomization reduction” (MPAR) to fabricate Fe‐based magnetic nanobundles. Part of the preparation process was shown in Figure S4 (Supporting Information). First step: FeCo nanobundles were synthesized by a reduction reaction as described previously,^[^ ^34^ ^]^ the difference was that the method of adding NaBH_4_ was changed from dropwise to atomization. Typically, FeCl_3_·6H_2_O (0.125 g), CoCl_2_·6H_2_O (0.104 g), and PVP (1 g) were dissolved in DI water (40 mL) to form transparent solution A. NaBH_4_ (0.4 g) was dissolved in DI water (40 mL) to prepare solution B. Afterward, solution A was placed on the permanent magnet ≈0.15 T and then kept spraying solution B into solution A. After the reaction, the black chain precipitates were collected by a magnet and washed alternately three times with ethyl alcohol and DI water. Second step: 50 mg of FeCo nanobundles were dispersed in a mixed solution containing 80 mL of ethyl alcohol and 16 mL of DI water in a beaker. Then, 2.0 g of concentrated ammonia solution (28 wt.%) was added and the mixed dispersion was mechanically stirred (500 rpm) for 10 min at 30 °C. After that, the stirring speed was decreased to 300 rpm, and 1 mL of TEOS was injected into the solution for prehydrolysis of TEOS. After the reaction for 4 h, the FeCo nanobundles could be uniformly coated by a layer of SiO_2_, and the obtained FS was collected by a magnet and washed with ethanol three times.

### Fabrication of ANFs/MXene/FeCo@SiO_2_ Nanobundles Aerogels (AMFS)

The aerogel was fabricated by the ice crystal template method. A certain proportion of Ti_3_CNT_x_ aqueous solution and ANFs aqueous dispersion was first added to a beaker, controlling the total solid content of 100 mg, and then stirred for 2 h. Subsequently, 10 mg of the FS was added and stirred evenly. The uniform suspension was frozen at −20 °C and freeze drying at −80 °C for 72 h, while the pressure was kept below 2 Pa. The aerogel with magnetic field orientation in the freezing process was named AMFS‐O, and the one without magnetic field orientation was named AMFS‐NO. According to the different mass ratios (x : y) of ANFs to MXene, the obtained AMFS‐O and AMFS‐NO aerogels could be labeled as AxMyFS‐O and AxMyFS‐NO, respectively. All aerogels containing FS were called AMFS for simplifying representations.

### Characterizations

The phase and crystal structure of samples were tested by X‐ray diffraction (XRD, DX‐2700) using Cu‐Ka radiation (*λ* = 1.54 Å). The morphology of samples was characterized by scanning electron microscopy (SEM, S‐4700, Hitachi, 15 kV, Japan) and transmission electron microscopy (TEM, JEM‐2100F, 200 kV, Japan). The magnetic properties of samples were measured by a vibrating sample magnetometer (VSM, manufactured by Lakeshore, Inc.). X‐ray photoelectron spectroscopy (XPS) was performed on a Thermo Scientific K‐Alpha spectrometer. Fourier transform infrared (FT‐IR) spectra were measured with a Thermo Scientific Nicolet iS5 spectrometer, with a resolution of 4.000 cm^−1^ in the range of 400–4000 cm^−1^. Electrical conductivities were detected by utilizing a standard four‐probe station (HPS2524). Surface temperature and infrared property at 120 °C were taken by a FLIR A325SC camera. The sample's mechanical properties were tested on a universal testing machine (Instron 5565, 5KN) with a loading rate of 5 mm min^−1^ at room temperature.

### CST Simulations

The electromagnetic field distribution and RCS simulation under actual far‐field response at 10 GHz were simulated by CST Studio Suite and all the mock objects were modeled based on real sizes. EW was generated as plane wave incidence along the Z direction. Corresponding simulation conditions were shown in Figure S1 (Supporting Information).

### Electromagnetic Measurements

Based on the wave‐guide method, the EW parameters (*ε*′, *ε*″, µ′, and µ″) of samples were measured over 8.2 – 12.4 GHz (X‐band) and 12.4 – 18.0 GHz (Ku‐band) in a vector network analyzer (VNA, 3672B‐S, Ceyear). The RL values of EW absorbers were calculated by the following transmission line equations:

(1) $$Z_{\mathrm{in}}=Z_{0}\sqrt{\frac{\mu_{r}}{\varepsilon_{r}}}\tanh\left(j\frac{2\pi fd}{c}\sqrt{\mu_{r}\varepsilon_{r}}\right)$$

(2) $$RL=20\mathrm{lg}\left|\frac{Z_{\mathrm{in}}-Z_{0}}{Z_{\mathrm{in}}+Z_{0}}\right|$$

where Z_in_ was the input impedance of the absorber, Z_0_ was the impedance of free space, µ′ and µ′′ were the real part and imaginary part of the complex permeability respectively, *ε*′ and *ε*′′ were the real part and imaginary part of the complex permittivity respectively, *ε*_r_ was the relative complex permittivity (*ε*_r_ = *ε*′ – j*ε*″), µ_r_ was the relative complex permeability (µ_r_ = µ′ – jµ″), *f* was the test frequency, *d* represents the thickness and *c* was the velocity of light. A suitable Z = Z_in_/Z_0_ value of close to 1 indicates a better impedance matching between space and absorber.

The EWAP was defined as an evaluation index, combined with density, thickness, and effective bandwidth, to further evaluate the EWA performance of tested aerogels. The EWAP values in X‐band were calculated by the following equation:

(3) $$\mathrm{EWAP}\left(\mathrm{GHz}\cdot {\mathrm{cm}}^{2}\cdot g^{-1}\right)=\frac{EAB}{\rho \times d}$$

where EAB was the effective absorption bandwidth (RL ≤ ‐10 dB), *ρ* was the bulk density of the aerogel, and *d* was the matching thickness.

## Conflict of Interest

The authors declare no conflict of interest.

## Supporting information

Supporting Information

## Data Availability

Research data are not shared.
