# Supplementary material for: Ordered Heterostructured Aerogel with Broadband Electromagnetic Wave Absorption Based on Mesoscopic Magnetic Superposition Enhancement
Source: Adv Sci (Weinh). 2023 May 7;10(21):2301599. doi: 10.1002/advs.202301599 (PMC10375159; doi:10.1002/advs.202301599)
Supplement: Supplementary file 1 — Supporting Information [file ADVS-10-2301599-s001.pdf]

ADVANCED SCIENCE
Open Access

Supporting Information

for *Adv. Sci.*, DOI 10.1002/advs.202301599

Ordered Heterostructured Aerogel with Broadband Electromagnetic Wave Absorption Based on Mesoscopic Magnetic Superposition Enhancement

Haojie Jiang, Lei Cai, Fei Pan, Yuyang Shi, Jie Cheng, Yang Yang, Zhong Shi, Xiaoli Chai, Hongjing Wu and Wei Lu**

Supporting Information

Ordered Heterostructured Aerogel with Broadband Electromagnetic Wave Absorption based on Mesoscopic Magnetic Superposition Enhancement

Haojie Jiang, Lei Cai, Fei Pan, Yuyang Shi, Jie Cheng, Yang Yang, Zhong Shi, Xiaoli Chai, Hongjing Wu, and Wei Lu**

The calculation of aerogel density:

The density of the aerogel is calculated by Equation S1. In detail, the length (l), width (w), and height (h) of cuboid aerogel were measured with vernier caliper and measuring its mass by electronic balance, which is accurate to 0.0001g. AMFS-O aerogels here deliver low density of 0.005 ~ 0.01 g/cm^3.

$$\rho=\frac{m}{l\times w\times h} \quad (1)$$

Chemical reaction equations for the preparation materials:

$$Fe^{3+}+Co^{2+}+5BH_4^-+15H_2O\rightarrow\left(Fe-Co^0\right)\downarrow+5B\left(OH\right)_3+17.5H_2\uparrow \quad (2)$$

$$Si\left(OC_2H_5\right)_4+4H_2O\rightarrow Si\left(OH\right)_4+4C_2H_5OH \quad (3)$$

$$Si\left(OH\right)_4\rightarrow SiO_2+2H_2O \quad (4)$$

Snoek limit and its modified formulas:

For most isotropic magnets, the microwave magnetic properties of the randomly distributed particles can be expressed as Equation S5, which is named by the Snoek limit [1]

$$\left(\mu_i-1\right)f_r=\frac{2\gamma}{3}4\pi M_s \quad (5)$$

μ_i, f_r, γ and $4\pi M_s$ are initial permeability, resonance frequency, gyromagnetic ratio, and

saturation magnetization, respectively. For this kind of material, the magnetic permeability drops rapidly to a very small value when the frequency reaches GHz frequency. However, when an easy magnetization plane exists in the material, its high-frequency property is expressed by Equation S6 [1a, 2]:

$$(\mu_i - 1) f_r = \frac{\gamma}{2} 4\pi M_s \sqrt{\frac{H_\theta}{H_\varphi}} \tag{6}$$

H_θ and H_φ are out-of-plane anisotropy fields and in-plane anisotropy fields. Normally, H_θ is always much larger than H_φ for the soft magnetic materials with planar anisotropy. Considering the influence of shape factors on the high-frequency permeability of single-domain particles, literature proposed the modified polar limit formula as Equation S7[3]:

$$(\mu_i - 1) f_r^2 = (\gamma 4\pi M_s)^2 \left(1 - 3N_k + \frac{2H_k}{4\pi M_s}\right) \tag{7}$$

N_k is the demagnetization factor along the easy magnetization axis of particles. H_k is an anisotropic field. It can be proved by Equation S7 that particles with strong anisotropy and small demagnetization factor in the direction of easy magnetization can obtain better permeability in the GHz frequency band.

CST simulation:

Electromagnetic Energy Density:

·Electromagnetic waves fluctuate in a sinusoidal manner. When the electric field has a maximum strength, so does the magnetic field. Both are zero at the same instant.

·The energy carried by the wave fluctuates in the same manner.

·A way to quantify the energy of an electromagnetic wave is to measure the total energy density carried by an electromagnetic wave (energy per unit volume).

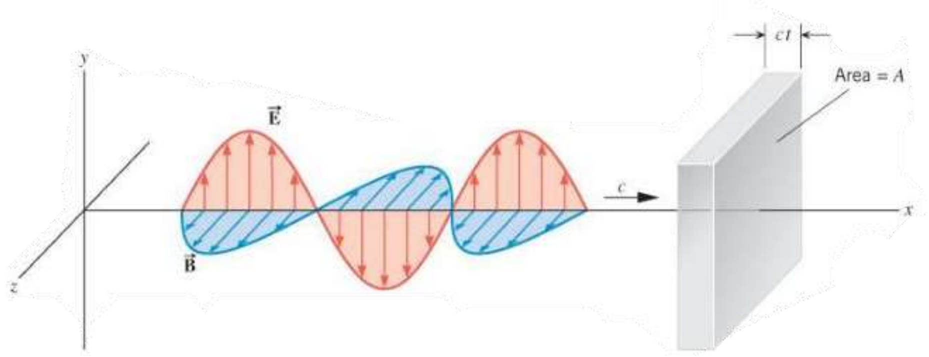

The following information is from the internal help file of CST software:

Energy within magnetic materials is a consequence of complex microscopic interactions, and which can't be modeled by FEM. The numerical computation of the magnetic energy relies on the behavior law of the considered material. If no persistent magnetic field is considered, for linear or nonlinear materials, the magnetic energy density Ψ is defined as:

$$\psi = \int_0^B H(B)\,dB \qquad (8)$$

$$\phi = \int_0^H B(H)\,dB \qquad (9)$$

The magnetic energy and coenergy densities can be computed using the B(H) curve defined within the numerical model. They represent the following areas:

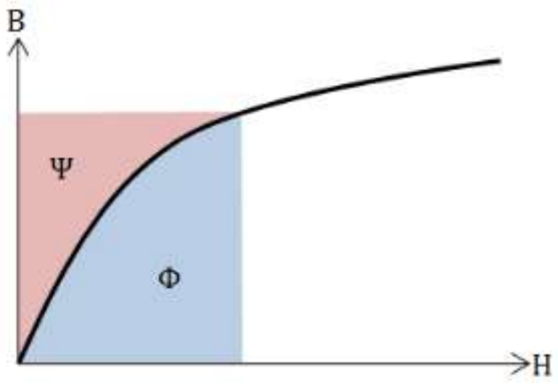

The sum of these two energies is defined as the product between B and H:

$$\psi + \phi = B \cdot H \qquad (10)$$

The total magnetic energy and coenergy are computed integrating Φ and Ψ throughout the whole domain:

$$\phi=\psi=\frac{1}{2}\frac{B^2}{\mu}=\frac{1}{2}\mu H^2 \tag{11}$$

The simulation was carried out by using CST microwave studio suite. Metamaterial-full structure workflow was chosen for simulation. Frequency domain solver was used in this simulation process. The overall properties of this simulation are shown in **Figure S1**.

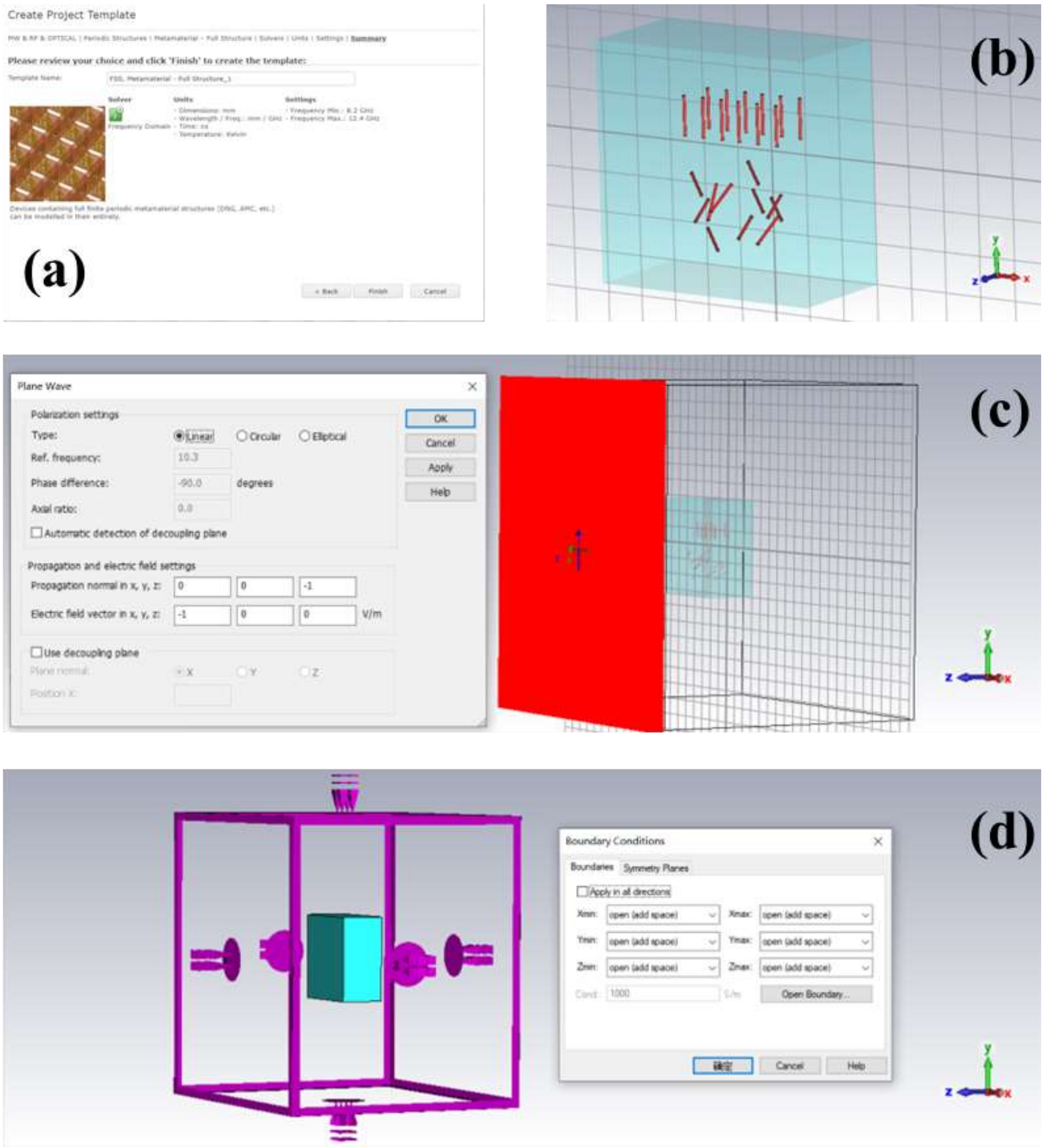

Figure S1. (a) The overall parameters of the simulation project. (b) The model of ordered FS and disordered FS structure (red) dispersed in A7M3 aerogel (bule) in CST microwave studio. (c) The set up of plane wave field source. (d) The setup of boundary conditions.

Radar equation and radar cross section (RCS) simulation theory:

Thus, RCS can be intuitively understood as the characteristic of the target object itself, which has nothing to do with distance R. Besides, the dimension of RCS is area (m^2).

$$\sigma\left(m^2\right)=\lim_{R\to\infty}4\pi R^2\left(\left|\frac{E_S}{E_i}\right|\right)^2=\lim_{R\to\infty}4\pi R^2\left(\left|\frac{H_S}{H_i}\right|\right)^2=\lim_{R\to\infty}4\pi R^2\frac{S_S}{S_i} \quad (12)$$

Here, E_s and E_i are the intensities of the scattered electric field and incident electric field, respectively. H_s and H_i stand for the intensities of the scattered magnetic field and incident magnetic field, and Ss and Si represent the power density of the scattered field and incident field. In addition, as explained in the Supporting Information, the decibel square meter unit (dB m^2) is typically used to measure radar cross section, where σ (dB m^2) = 10 log $\sigma(m^2)$. Due to the dynamic range of the radar cross section of the target is very large, so it is often expressed by decibel square meters (dB m^2):

$$\sigma\left(dBm^2\right)=10\log\sigma\left(m^2\right) \quad (13)$$

Other supporting materials：

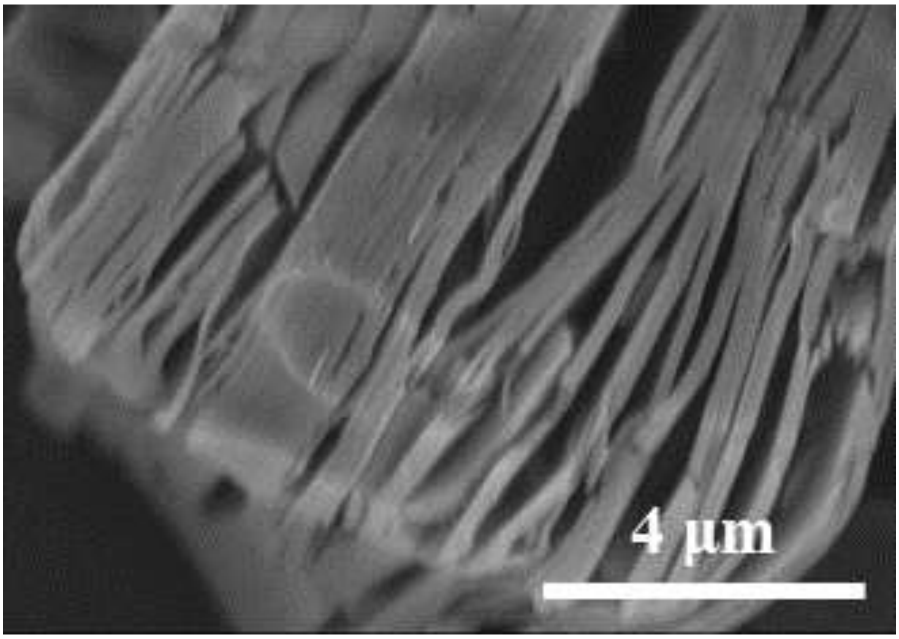

Figure S2. SEM images of exfoliated m-Ti_3CNT_x MXene.

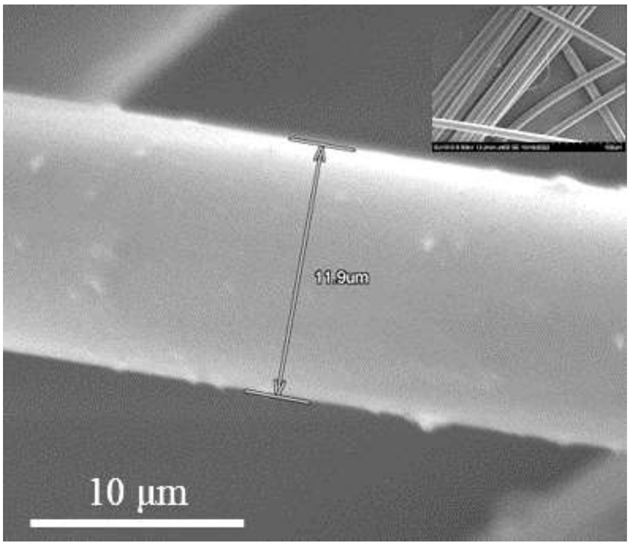

Figure S3. SEM images of original aramid fibers.

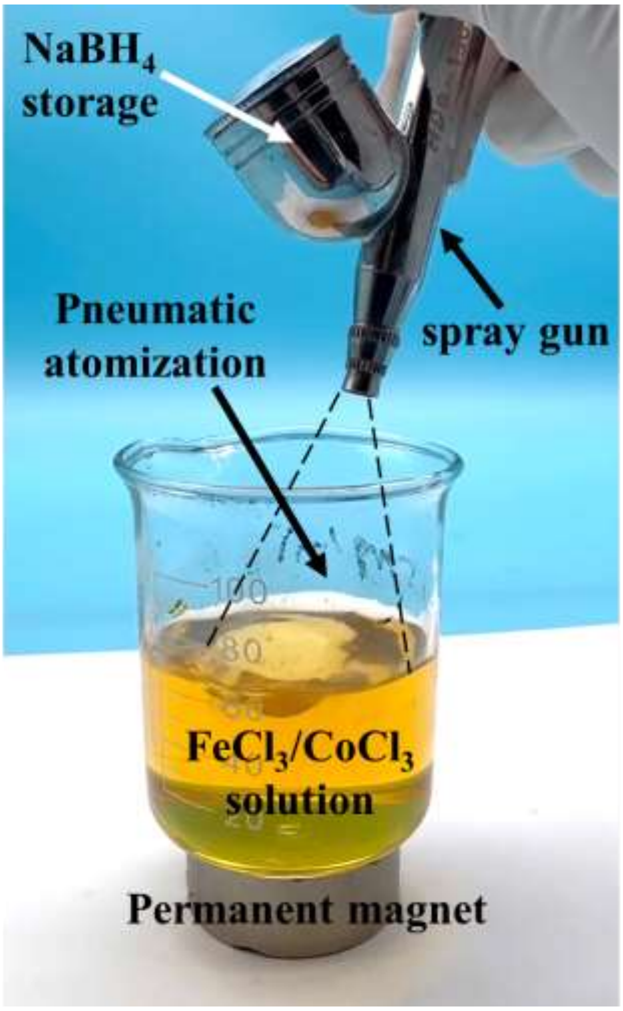

Figure S4. Magnetic field induced pneumatic atomization reduction" (MPAR) to fabricate Fe-based magnetic nanobundles.

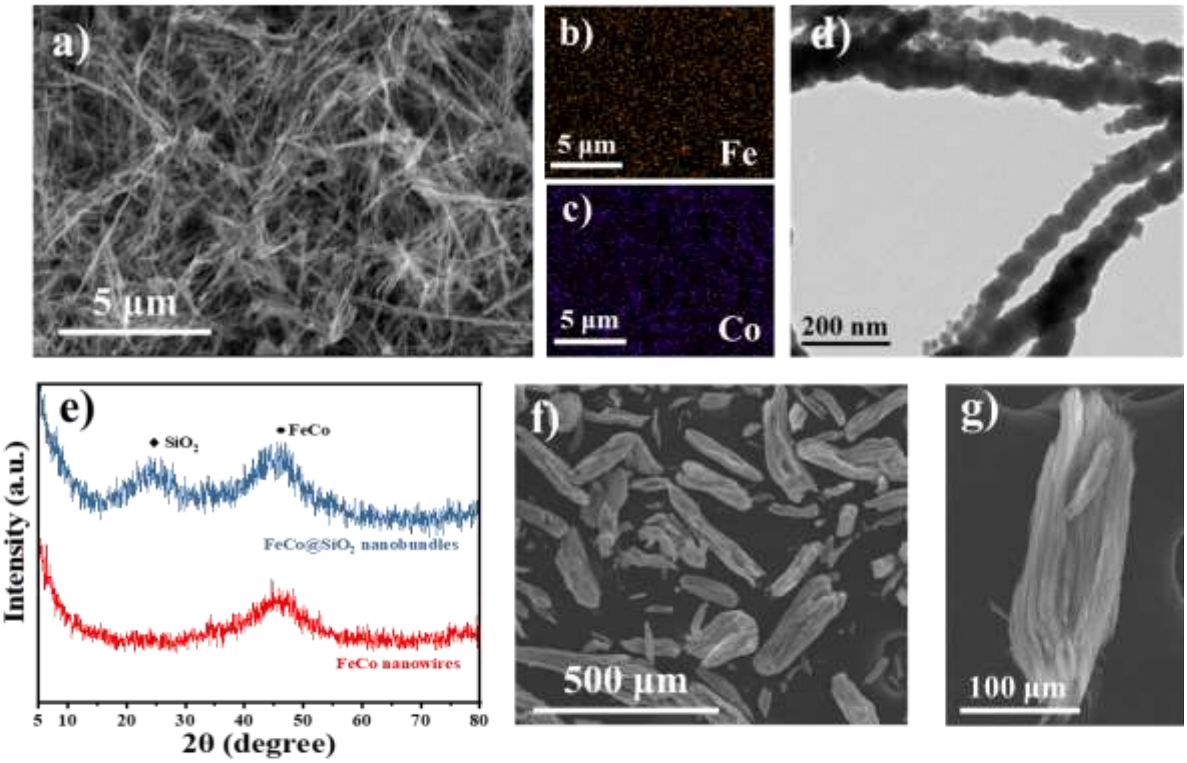

Figure S5. SEM image of (a) FeCo nanowires. Elemental mapping images of (b) Fe, (c) Co corresponding to (a). (d) TEM image of FeCo nanowires. (e) XRD patterns of FeCo@SiO_2 nanobundles and FeCo nanowires. (f, g) SEM image of nanobundles.

we have analyzed the elemental composition of FeCo nanowires by EDS, as shown in **Figure S5**. The EDS images exhibit the uniform existence of Fe and Co in **Figure S5b, c**. On the other hand, it can be found that the diameter of the prepared FeCo wire is below 100 nm by TEM in **Figure S5d**, which can be recognized as nanowire. Furthermore, the XRD pattern of FeCo nanowires in **Figure S5e** reveal the similar structure with FeCo@SiO_2 nanobundles. Therefore, the element composition, TEM analysis, and phase analysis illustrate the successful synthesis of FeCo nanowire.

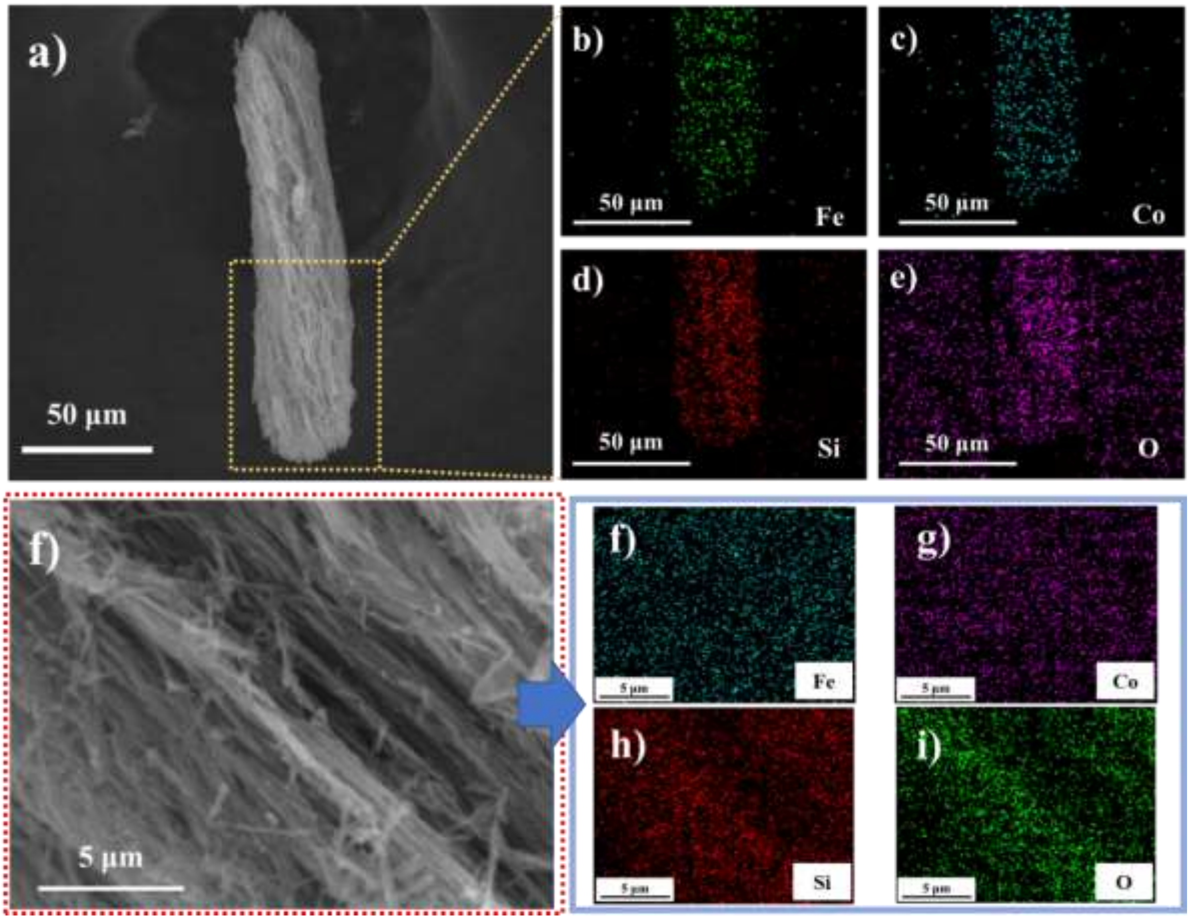

Figure S6. (a) SEM image of FS. Elemental mapping images of (b) Fe, (c) Co, (d) Si, and (e) O corresponding to (a). (f) SEM image of enlarged FS. Elemental mapping images of (f) Fe, (g) Co, (h) Si, and (i) O corresponding to (f).

we have analyzed the elemental composition of FeCo@SiO2 nanobundles by EDS, as shown in **Figure S6**. The EDS images exhibit the uniform existence of Fe, Co, Si, and O (including the oxygen element of the conductive tape), which illustrates the successful synthesis of FeCo@SiO2 nanobundles.

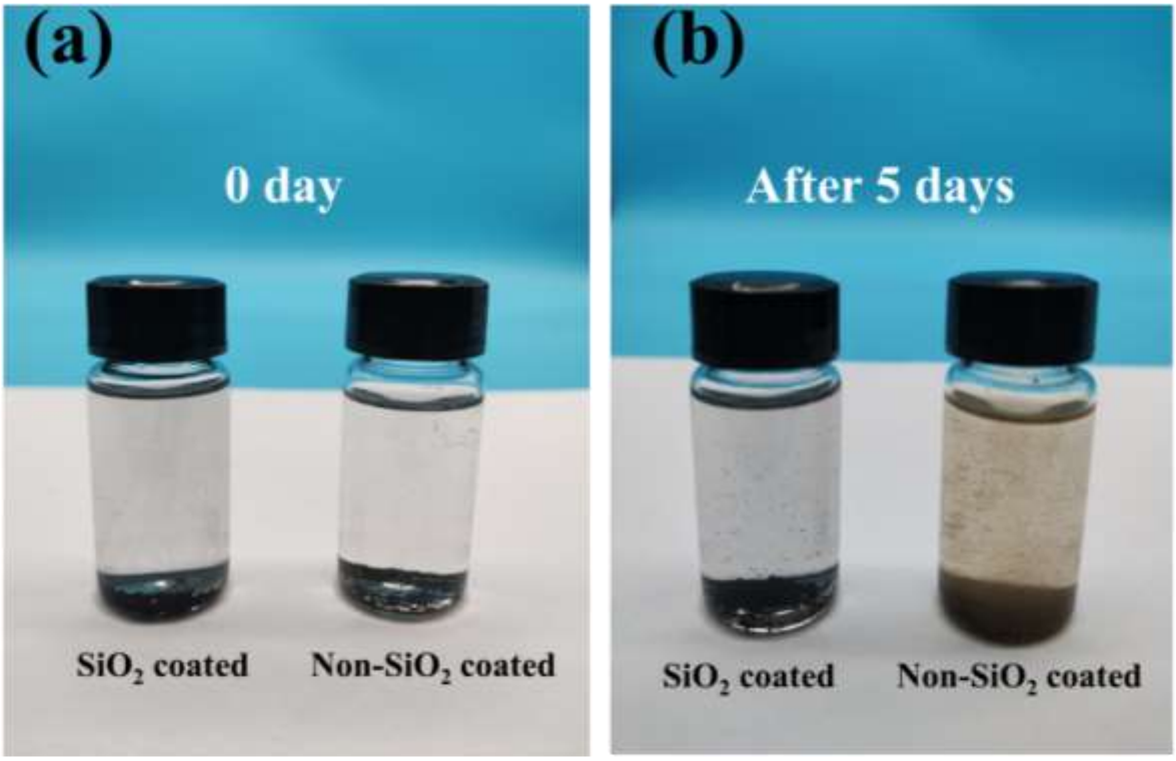

Figure S7 (a) Original state of SiO_2 coated FS and non-SiO_2 coated FS in water. (b) State of SiO_2 coated FS and non-SiO_2 coated FS in water after 5 days.

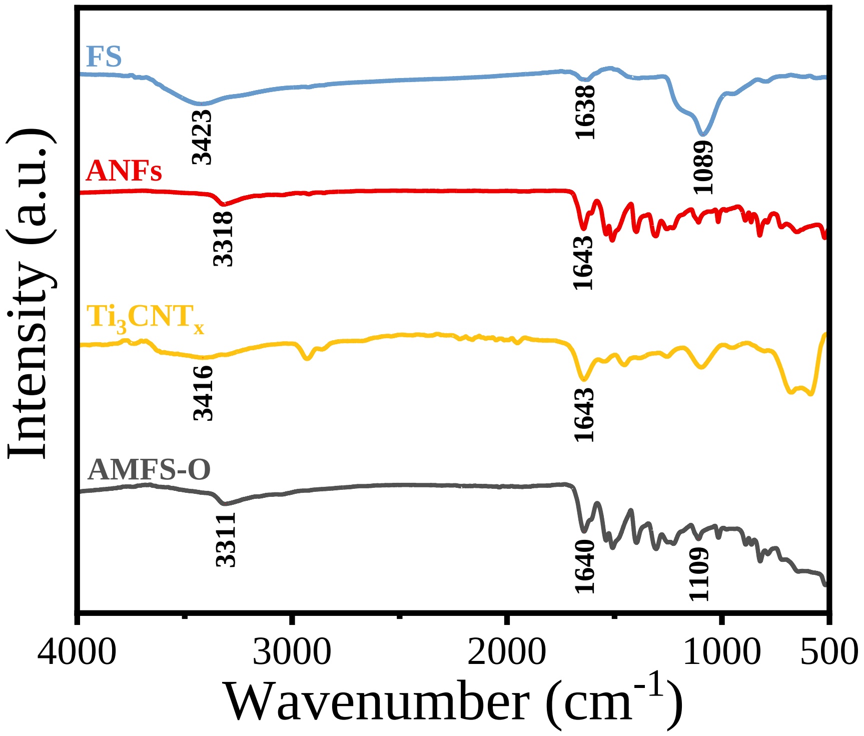

Figure S8. FTIR spectra of FS, ANFs, Ti_3CNT_x, and AMFS-O.

Figure S8 shows the FTIR spectra of ANFs, Ti_3CNT_x MXene, FS, and A7M3FS-O aerogel.

The FS has several chemical signature peaks at 3423 cm^{-1}, 1638 cm^{-1}, and 1089 cm^{-1} assigned to the stretching vibration of a hydroxyl group (−OH) from the adsorbed water molecule, the stretching vibration of the carbonyl group (C=O) of PVP and asymmetric vibrations of O-Si-O[4]. The FTIR spectrum of ANFs presents the band at 3318 cm^{-1} and 1643 cm^{-1} corresponding to the stretching vibration of the carbonyl group (C=O) and the stretching vibration of N−H, respectively[5]. The FTIR spectrum of Ti_3CNT_x MXene presents the bands at 3416 cm^{-1} and 1643 cm^{-1} corresponding to the vibration absorption of the hydroxyl group (−OH) and C=O, respectively. For the A7M3FS-O aerogel, the FTIR spectrum of which shows no obvious changes of the characteristic peaks. By contrast, the characteristic peak of the peaks of N-H and C=O bonds shifted to 3311 cm^{-1} and 1640 cm^{-1}, so the redshift of the characteristic peak confirmed the presence of hydrogen bonds[6]. This is due to the influence of the chemical environment around the C=O bond by the groups enriched on the surface of MXene (such as-OH, -F) and the carbonyl group acting as the hydrogen bond receptor, which led to the deviation of the characteristic peak[7]. These results illustrate that no obvious oxidization of Ti_3CNT_x occurred during the assembly process, accompanied by a favorable combination owing to hydrogen bonding and robust ANFs networks, thus guaranteeing the final mechanic properties and EWA performance of A7M3FS-O aerogels.

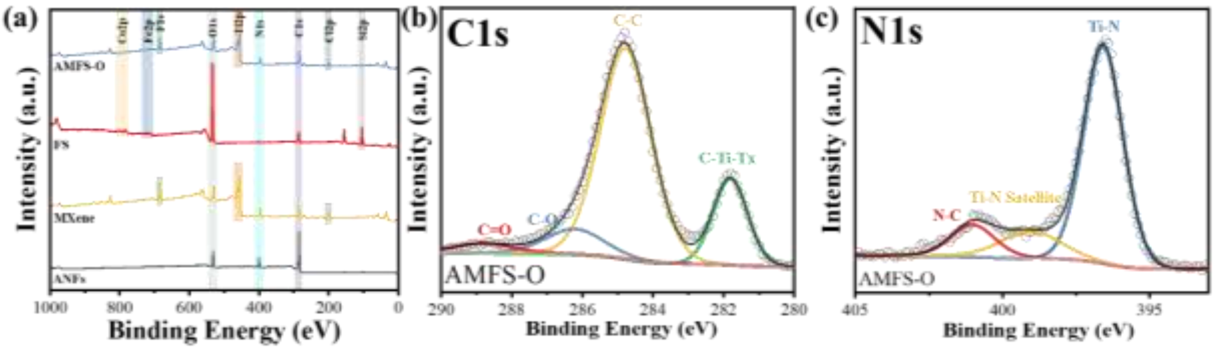

Figure S9. (a) XPS wide-scan spectra of AMFS-O, FS, pristine Ti_3CNT_x, and ANFs. (b) C 1s and (c) N1s high-resolution XPS spectra of AMFS-O.

X-ray photoelectron spectroscopy (XPS) was carried out to demonstrate the surface

chemical structures of pristine materials and synthetic aerogel. As confirmed by XPS wide-scan spectrum, the Ti, C, N, O, Cl, and F signals in the AMFS-O reveal the successful assembly of Ti_3CNT_x, indicating a plethora of polar groups attach to the surface of Ti_3CNT_x flakes, including −O, −Cl, −OH, and −F. Subsequently, the appearance of characteristic peaks for Fe 2p, Co 2p, and Si 2p further proves the combination of FS. The high-resolution Ti 2p spectra (**Figure 1o**) is deconvolved into two peaks (Ti $2p_{3/2}$, Ti $2p_{1/2}$), indicating the presence of TiO_2 $2p_{3/2}$ (458.8 eV), C-Ti-Tx $2p_{3/2}$ (457.24 eV), Ti-N $2p_{3/2}$ (456 eV), and Ti-C $2p_{3/2}$ (455 eV) peaks. Simultaneously, larger peaks of TiO_2 in Ti 2p and O 1s spectra (**Figure 1p**) are detected in the composite sample than that in pure MXene. The mild oxidation may result from ultrasound, agitation, and slight dissociative Fe^{3+}. The peak of H_2O in O 1s may be caused by incomplete drying before the test. Since the surface of FS is coated with SiO_2, the peak of SiO_2 (532.70 eV) appears in the O 1s of AMFS-O. Meanwhile, characteristic peaks at 218.7 eV (C-Ti-Tx), 284.8 eV (C-C), 286.2 eV (C-O), and 288.8 eV (C=O) are observed in the high-resolution C 1s spectra of samples. Moreover, N-C (401.05 eV), Ti-N satellite (399.00 eV), and Ti-N (396.57 eV)1s also confirmed the successful doping of the N element in MXene.

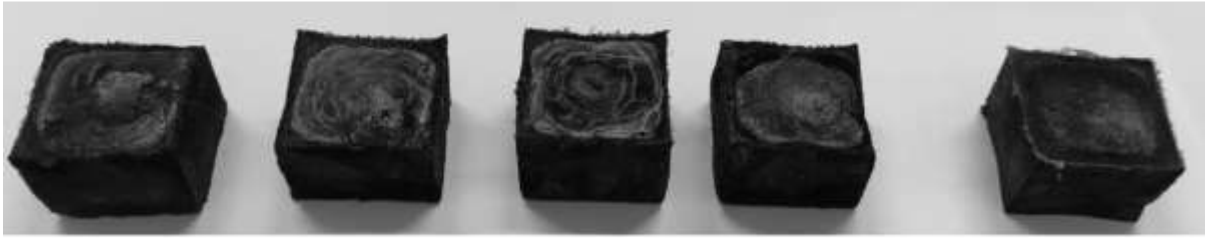

Figure S10. The optical photograph of samples.

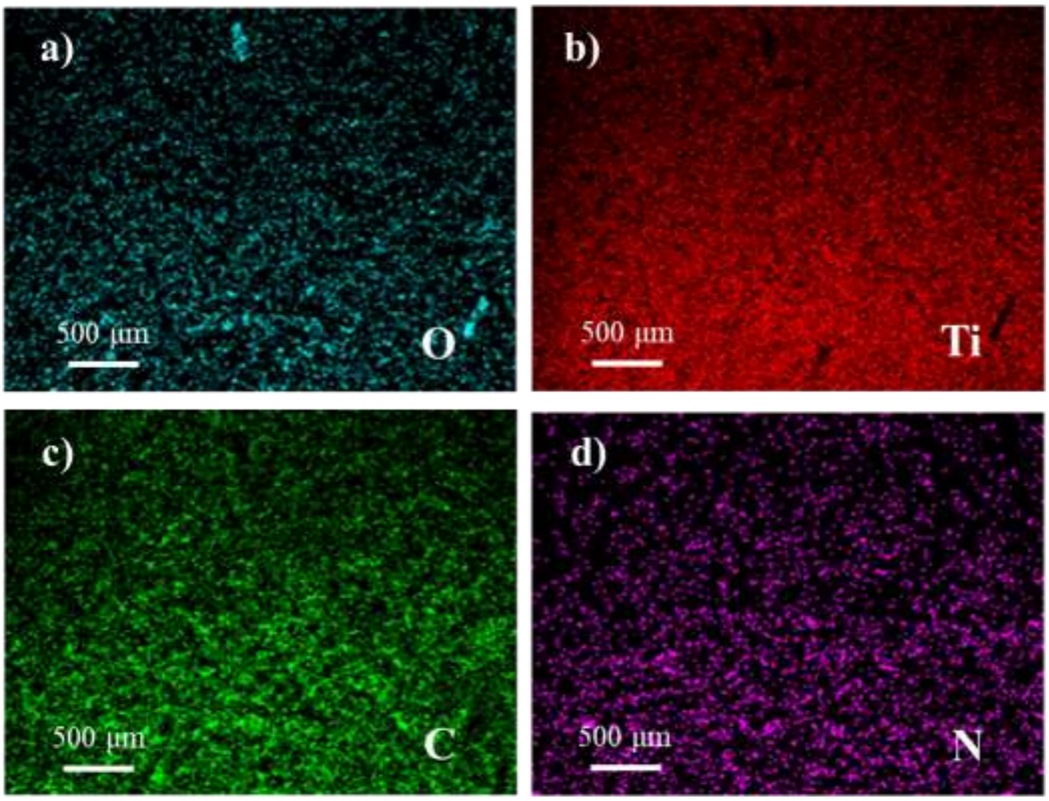

Figure S11. Corresponding elemental mapping images of (a) O, (b) Ti, (c) C, (d) N corresponding to **Figure 2**g.

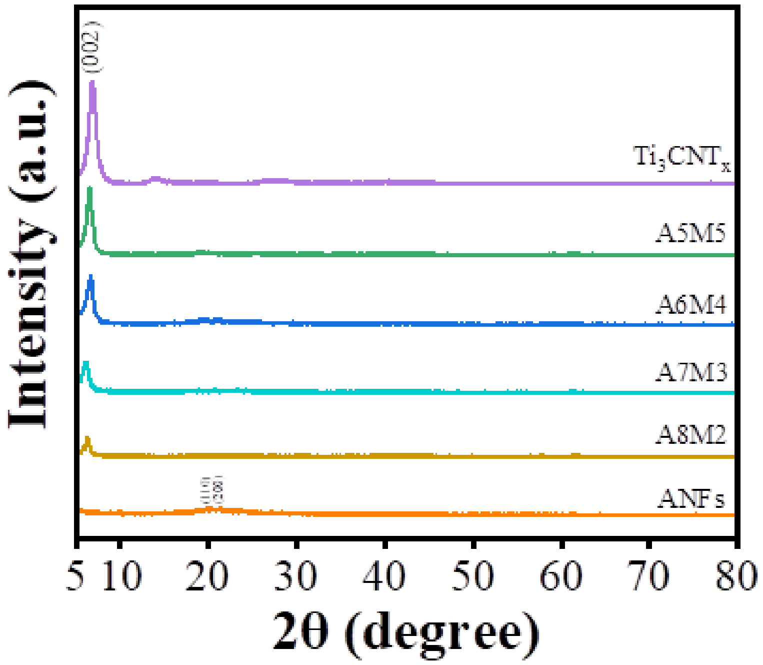

Figure S12. XRD patterns of Ti_3CNT_x, AM aerogels of different compositions, and ANF aerogel.

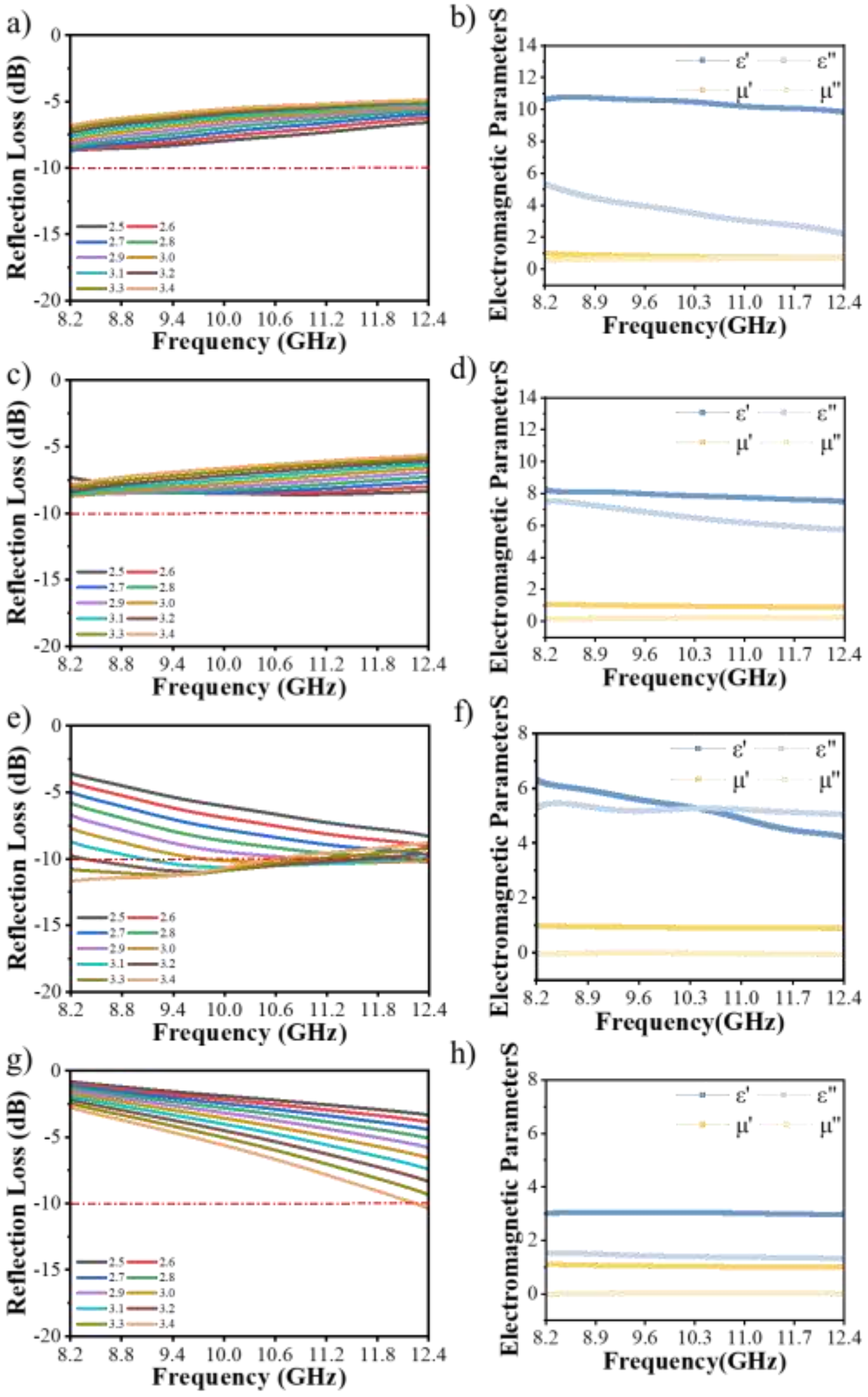

Figure S13. RL curves of (a) A5M5, (c)A6M4, (e) A7M3, (g) A8M2 composite aerogel, and electromagnetic parameters of (b) A5M5, (d)A6M4, (f) A7M3, (h) A8M2 composite aerogel.

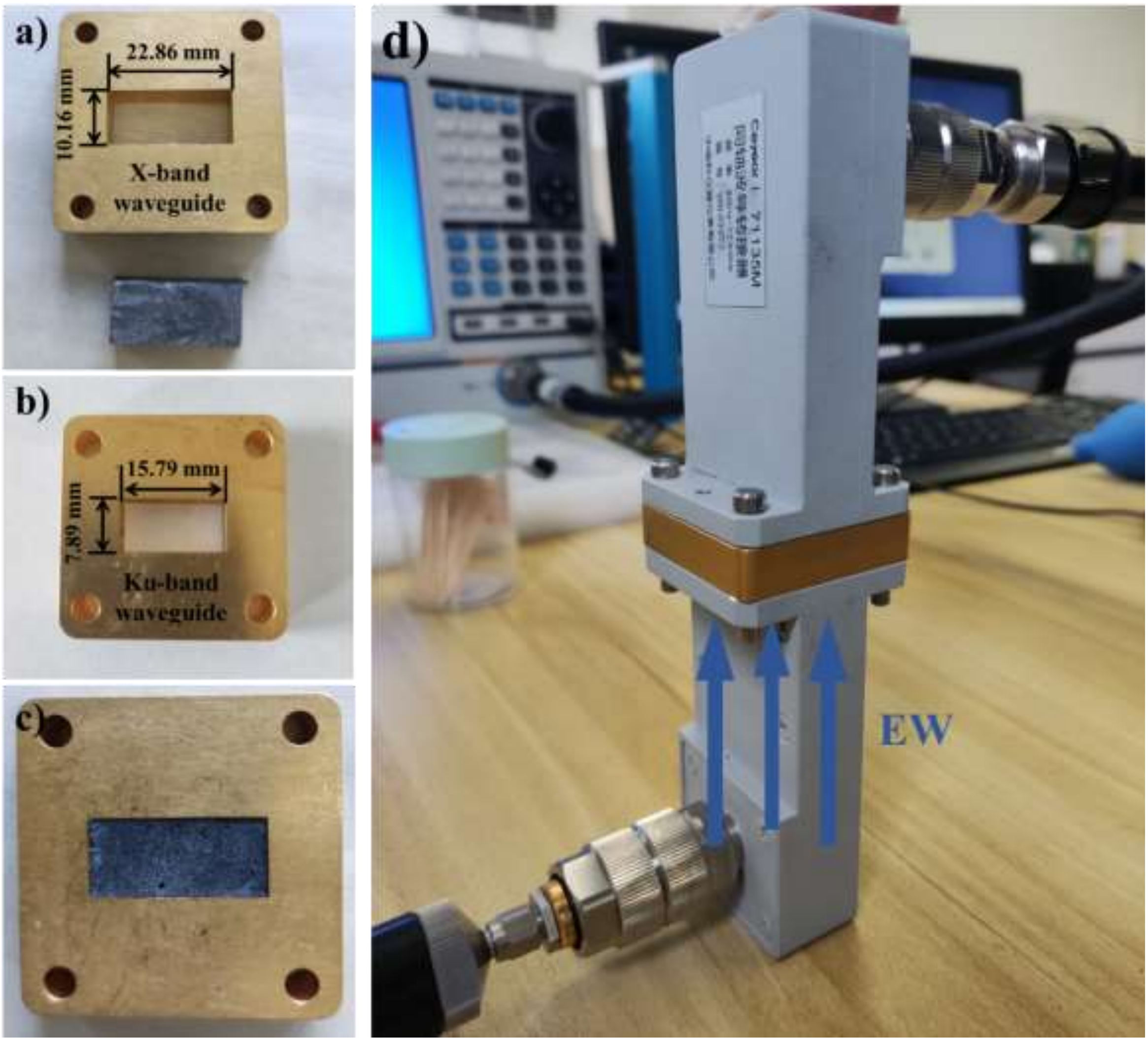

Figure S14. (a) The sizes of the X-band waveguide mold and the cut aerogel to be tested. (b) The sizes of the Ku-band waveguide mold. (c) The X-band waveguide mold with sample embedded inside. (d) The test image of the wave guide method.

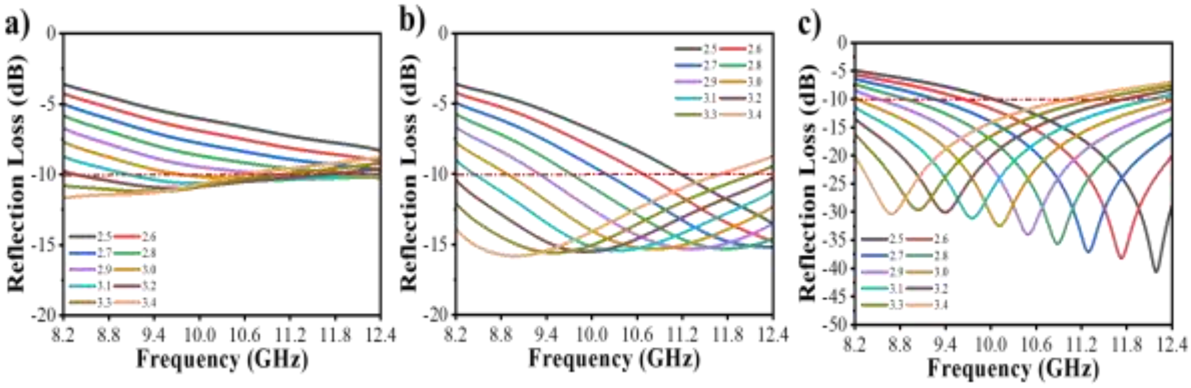

Figure S15. The RL curves with different thicknesses of samples (a) A7M3, (b) A7M3FS-NO,

and (c) A7M3FS-O.

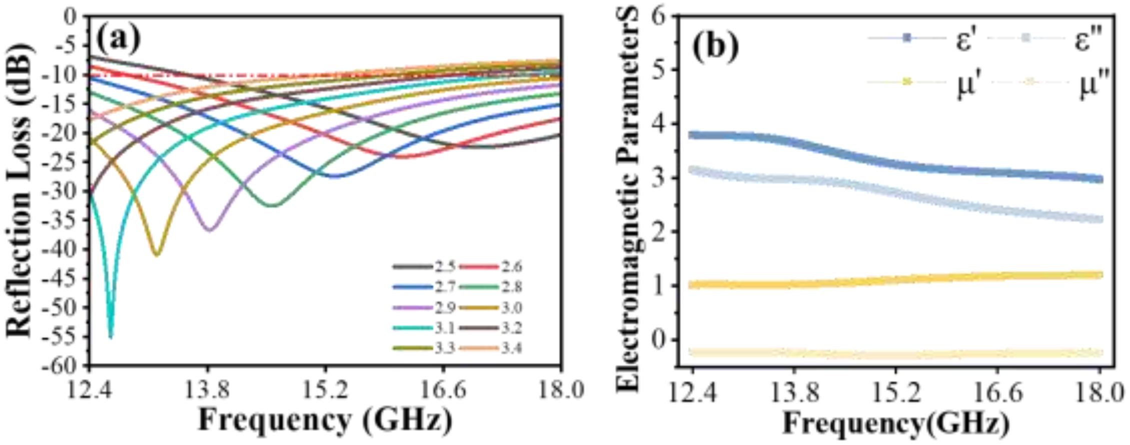

Figure S16. (a) The RL curves with different thicknesses and (b) electromagnetic parameters of A7M3FS-O at Ku-band.

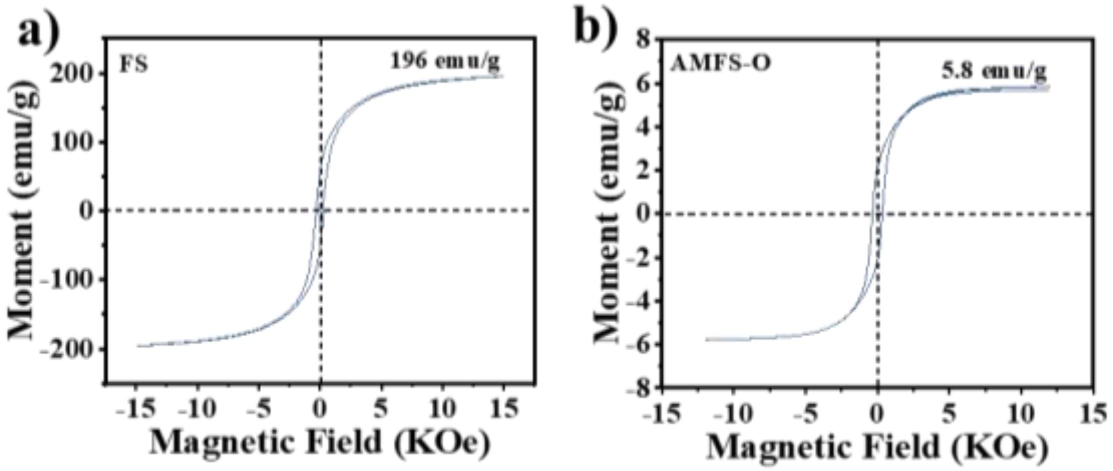

Figure S17. Hysteresis loops of as-prepared (a) FS and (b) AMFS-O.

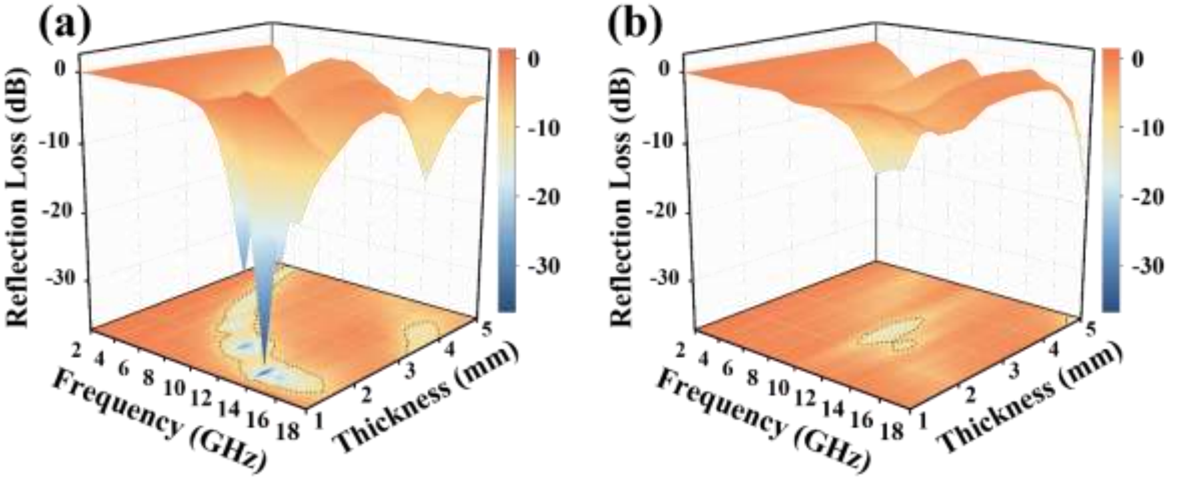

Figure S18 The 3D plots of RL values of (a) FS, and (b) FeCo nanowires.

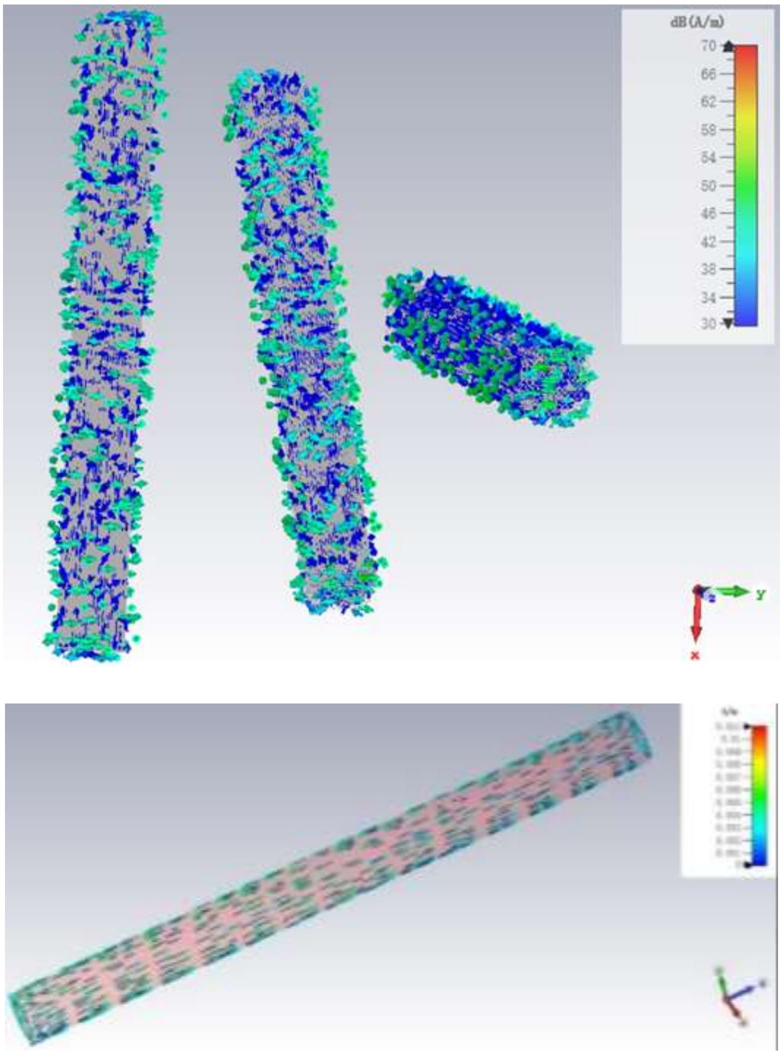

Figure S19. Simulation of surface currents of FS and single magnetic chain.

Table S1. EWA performance of representative magnetic Mxene-based absorbers and other magnetic-doped materials at the X-band

Samples	Matrix	RL ≤ -10dB EAB (GHz)	RL ≤ -10dB d (mm)	Density (g/cm^3)	EWAP ($GHz \cdot cm^2 \cdot g^{-1}$)	Refs.
Fe-doped $CeOHCO_3$	paraffin	3.2	2.4	> 0.9	14.8	[8]
g-C_3N_4@Fe@C	paraffin	3.0	1.8	> 0.9	18.5	[9]
γ-Al_2O_3@Ni@C	paraffin	3.3	1.7	> 0.9	21.6	[10]
chiral PPy/Fe_3O_4	paraffin	4.2	2.0	> 0.9	23.3	[11]
$Ti_3C_2T_x$/Ni/Aluminosilicate Glass	AS glass	3.0	2.04	1.5	9.7	[12]
Ti_3CNT_x/Bacterial Cellulose /Ni Chains aerogel	paraffin	2.5	2.5	> 0.9	11.1	[13]
$Ti_3C_2T_x$/Ni flower	paraffin	2.1	2.0	> 0.9	11.6	[14]
$Ti_3C_2T_x$ /CNTs/Magnetic Nanospheres	paraffin	2.4	2.0	> 0.9	13.3	[15]
$Ti_3C_2T_x$/Ni	paraffin	3.5	2.5	> 0.9	15.6	[16]
3D Hierarchical Magnetic $Ti_3C_2T_x$ Network	paraffin	2.8	2.0	> 0.9	15.6	[17]
$Ti_3C_2T_x$/FeNi	paraffin	3.0	2.0	> 0.9	16.7	[18]
$Ti_3C_2T_X$/CNTs/Fe_3O_4	paraffin	3.2	2.0	> 0.9	17.8	[19]
$Ti_3C_2T_x$/PMMA/Ni	paraffin	3.2	2.0	> 0.9	17.8	[20]

Microspheres						
$Ti_3C_2T_x$ /Fe_3O_4 Microspheres	paraffin	4.2	2.5	> 0.9	18.7	[21]
$Ti_3C_2T_x$/FeCo	paraffin	3.2	1.6	> 0.9	22.2	[22]
$Ti_3C_2T_x$ /Graphene/FeS foam	paraffin	4.2	2.0	> 0.9	23.3	[23]
$Ti_3C_2T_x$/Ni Chain/ZnO	Cotton fabric	4.2	2.2	> 0.3	63.6	[24]
$Ti_3C_2T_x$/Graphene/Ni Aerogel	PDMS	4.2	3.5	> 0.097	123.7	[25]
**A7M3FS-O**	**Air**	**4.2**	**3.0**	**0.01**	**1400**	**This work**

Densities of pure wax, PDMS, and epoxy were 0.9, and 0.097 g/cm^3, respectively.

References

[1] a) Y. Zhang, P. Wang, T. Ma, Y. Wang, L. Qiao, T. Wang, *Appl. Phys. Lett.* **2016**, 108; b) J. L. Snoek, *Physica* **1948**, 14, 207.

[2] K. N. Rozanov, Z. W. Li, L. F. Chen, M. Y. Koledintseva, *J. Appl. Phys.* **2005**, 97.

[3] a) R. K. Walser, W. Win, P. M. Valanju, *IEEE Transactions on Magnetics* **1998**, 34, 1390; b) O. Acher, A. L. Adenot, *Phys. Rev. B* **2000**, 62, 11324.

[4] T. Gholami, M. Salavati-Niasari, M. Bazarganipour, E. Noori, *Superlattices and Microstructures* **2013**, 61, 33.

[5] M. Yang, K. Cao, L. Sui, Y. Qi, J. Zhu, A. Waas, E. M. Arruda, J. Kieffer, M. D. Thouless, N. A. Kotov, *ACS Nano* **2011**, 5, 6945.

[6] Z. Ma, S. Kang, J. Ma, L. Shao, Y. Zhang, C. Liu, A. Wei, X. Xiang, L. Wei, J. Gu, *ACS Nano* **2020**, 14, 8368.

[7] Y. Du, J. Xu, J. Fang, Y. Zhang, X. Liu, P. Zuo, Q. Zhuang, *Journal of Materials Chemistry A* **2022**, DOI: 10.1039/d1ta11025j.

[8] X. Wang, F. You, X. Wen, K. Wang, G. Tong, W. Wu, *Chemical Engineering Journal* **2022**, 445, 136431.

[9] B. Fan, L. Xing, K. Yang, F. Zhou, Q. He, G. Tong, W. Wu, *Chemical Engineering Journal* **2023**, 451, 138492.

[10] K. Fu, X. Liu, Y. Yang, Z. Wang, W. Zhou, G. Tong, X. Wang, W. Wu, *Chemical Engineering Journal* **2023**, 457, 141318.

[11] X. Yang, B. Fan, X. Tang, J. Wang, G. Tong, D. Chen, J. Guan, *Chemical Engineering Journal* **2022**, 430, 132747.

[12] W. Luo, M. Wang, K. Wang, P. Yan, J. Huang, J. Gao, T. Zhao, Q. Ding, P. Qiu, H. Wang, P. Lu, Y. Fan, W. Jiang, *Adv Sci (Weinh)* **2022**, 9, e2104163.

[13] F. Pan, Y. Rao, D. Batalu, L. Cai, Y. Dong, X. Zhu, Y. Shi, Z. Shi, Y. Liu, W. Lu, *Nanomicro Lett* **2022**, 14, 140.

[14] H. Cheng, Y. Pan, X. Wang, C. Liu, C. Shen, D. W. Schubert, Z. Guo, X. Liu, *Nano-Micro Lett.* **2022**, 14, 63.

[15] C. Zhang, Z. Wu, C. Xu, B. Yang, L. Wang, W. You, R. Che, *Small* **2022**, 18.

[16] X. Li, W. You, L. Wang, J. Liu, Z. Wu, K. Pei, Y. Li, R. Che, *ACS Applied Materials and Interfaces* **2019**, 11, 44536.

[17] J. Xu, W. Ma, P. He, Y. Zhou, X. Liu, Y. Chen, P. Zuo, Q. Zhuang, *Journal of Materials Chemistry A* **2023**, 11, 330.

[18] J. He, X. Liu, Y. Deng, Y. Peng, L. Deng, H. Luo, C. Cheng, S. Yan, *Journal of Alloys and Compounds* **2021**, 862.

[19] C. Zhang, Z. Wu, C. Xu, B. Yang, L. Wang, W. You, R. Che, *Small* **2022**, 18, e2104380.

[20] C. Wen, X. Li, R. Zhang, C. Xu, W. You, Z. Liu, B. Zhao, R. Che, *ACS Nano* **2021**, DOI: 10.1021/acsnano.1c08957.

[21] X. Li, M. Zhang, W. You, K. Pei, Q. Zeng, Q. Han, Y. Li, H. Cao, X. Liu, R. Che, *ACS Applied Materials and Interfaces* **2020**, 12, 18138.

[22] J. He, D. Shan, S. Yan, H. Luo, C. Cao, Y. Peng, *Journal of Magnetism and Magnetic Materials* **2019**, 492.

[23] S. Li, X. Tang, X. Zhao, S. Lu, J. Luo, Z. Chai, T. Ma, Q. Lan, P. Ma, W. Dong, Z. Wang, T. Liu, *J. Mater. Sci. Technol.* **2023**, 133, 238.

[24] S. Wang, D. Li, Y. Zhou, L. Jiang, *ACS Nano* **2020**, 14, 8634.

[25] L. Liang, Q. Li, X. Yan, Y. Feng, Y. Wang, H. B. Zhang, X. Zhou, C. Liu, C. Shen, X. Xie, *ACS Nano* **2021**, 15, 6622.
